# DeSpotX: Identifiability-Based Decontamination for Spatial Transcriptomics

**DOI:** 10.64898/2026.05.12.724704

**Published:** 2026-05-14

**Authors:** Ruo Han Wang, Andrew J. Gentles

**Affiliations:** Stanford University

## Abstract

Spatial transcriptomics (ST) at single-cell resolution profiles gene expression in its native spatial context, but a substantial fraction of transcripts contaminate neighboring cells, compromising downstream biological analyses. Existing decontamination methods rely on heuristic priors and either ignore the spatial structure of contamination or aggregate over neighbors without separating contamination from native expression, leaving the decomposition ambiguous. To resolve this ambiguity, we introduce DeSpotX, a deep generative model that uses anchor genes, defined as genes not natively expressed in a given cell cluster, to constrain the contamination decomposition and make it identifiable. DeSpotX further uses spatial information to estimate contamination locally through a cluster-masked, distance-weighted average over neighboring cells, and prevents over-correction of low-expression signal through a learned diffusion prior. On spike-in simulations across five datasets and four ST platforms, DeSpotX achieves AUROC > 0.94 on every dataset, with gains of 0.02 to 0.12 over the best baseline, and remains robust to inaccuracies in the cell-cluster annotation and in anchor gene construction. On real tissues, we show that the decontaminated counts produce improved marker-gene specificity, more spatially coherent expression, and cell-cell communication networks consistent with known biology. We further show that iterating decontamination and cell-cluster annotation refines these outcomes, reassigning ligand-receptor signaling to the expected source cells in mouse brain and breast cancer tissues.

## 1 Introduction

Single-cell-resolution spatial transcriptomics (ST) platforms, such as Xenium [1], MERFISH [2, 3], CosMx [4], and Stereo-seq [5], now profile millions of cells in tissue, enabling the study of cellular organization, cell-cell communication, and disease microenvironments at single-cell resolution [6, 7]. However, these technologies suffer from a systematic contamination artifact, where transcripts from one cell are frequently assigned to neighboring cells through ambient diffusion, segmentation errors, and vertical overlap of cells in the tissue slice [8, 9, 10]. The contamination is widespread, with 20–40% of cells showing substantial contamination and 10–20% of cells expressing markers of mutually exclusive cell types according to recent studies [8, 9]. These artifacts compromise downstream biological analyses, causing misannotation of cell types, blurring the spatial organization of gene expression, and producing spurious cell-cell communication networks that can mislead the discovery of therapeutic targets.

Decontamination of ST data poses three fundamental challenges. First, the native and contamination components cannot be uniquely separated from observed counts. The same observed count distribution can arise from multiple combinations of native expression, contamination profile, and contamination fraction, so methods fail to identify which combination is correct without additional constraints on the decomposition [11]. Second, contamination in ST is spatially structured, since it diffuses locally from nearby cells and the contaminating signal reflects the cellular composition of each cell’s local environment. A single global ambient profile, as used by methods designed for dissociated single-cell RNA-seq [12, 13, 14], fails to capture this local structure. Third, low-expression genes are vulnerable to over-correction during contamination removal [15], even though they often carry informative biological signal. At low expression levels, even small errors in the contamination estimate are large relative to the true signal, so the subtraction can remove real expression along with contamination.

To address each of these challenges, we present DeSpotX (https://github.com/Gentles-lab/DeSpotX), an identifiability-based deep generative model for decontaminating single-cell-resolution ST data. For the *non-identifiability* challenge, DeSpotX introduces anchor genes, defined as genes that are not natively expressed in a given cell cluster and inferred automatically from per-cluster expression rates, and uses them to constrain the decomposition. For the *spatial structure* challenge, DeSpotX estimates the local contamination profile as a cluster-masked, distance-weighted average over cross-cluster spatial neighbors, separating spatially diffused contamination from each cell’s native expression. For the *signal preservation* challenge, DeSpotX places a learned diffusion prior over the latent state encoding native expression, regularizing recovered profiles toward learned cluster-conditioned distributions and preventing the noise in contamination estimates from over-correcting low-expression signal.

Across five spatial transcriptomics datasets spanning four platforms and four tissue contexts, DeSpotX outperforms all leading decontamination baselines and maintains strong performance even when cell-cluster annotations or anchor gene construction are imperfect. Downstream analyses on the decontaminated counts yield cleaner cluster separation, more spatially coherent marker expression, and more biologically coherent cell-cell communication networks. We further show that iterating between decontamination and cell-cluster annotation progressively improves these outcomes.

## 2 Related work

### Decontamination of single-cell RNA-seq.

Several methods correct ambient RNA contamination in dissociated single-cell RNA-seq. SoupX [13] estimates a global ambient profile from empty droplets and subtracts a per-cell scaled version. DecontX [12] fits a Bayesian mixture model that decomposes each cell’s expression into its native cluster profile and a global contamination profile aggregated across clusters, recovering per-cell contamination fractions by variational inference. CellBender [14] models raw barcode-level counts with a learned ambient prior estimated from empty droplets, separating ambient transcripts from cell-derived expression. These methods rely on a single global ambient profile and do not incorporate spatial information, making them unable to capture the spatial structure of contamination in single-cell-resolution ST.

### Decontamination of spatial transcriptomics.

Recent methods adapt count correction to ST data by incorporating spatial information into the contamination estimate. SpaceBender [10] replaces CellBender’s global ambient profile with a spatially local profile computed by averaging expression over k-nearest-neighbor or distance-based spatial neighbors. DenoIST [9] fits a Poisson mixture that classifies each gene count as endogenous or contamination based on the cell’s count relative to its local neighborhood. ResolVI [8] decomposes observed counts into native expression, neighbor-derived diffusion, and non-specific background. While these methods leverage spatial structure, none separate spatially diffused contamination from native expression at each location. Moreover, all of these methods regularize the decomposition through Bayesian priors, latent-variable architectures, or mixture-model assumptions, none of which resolve the underlying non-identifiability. These regularizers can produce decompositions that fit the observed counts, but the recovered decompositions can deviate from the true profile. A detailed comparison between DeSpotX and existing methods is provided in Appendix A.

### Identifiability of latent variable models.

Identifiability is a long-standing concern in latent variable models, where multiple parameter combinations can produce the same observed data distribution [11]. Two main strategies resolve non-identifiability. The first uses auxiliary variables to make latent dimensions conditionally independent, as in identifiable VAEs [11], but no such auxiliary variable is available in the decontamination setting. The second imposes structural constraints on the model, such as anchor-word identifiability in topic models [16] and separability in non-negative matrix factorization [17], and fits the decontamination problem naturally, since each cell cluster expresses only a subset of genes. DeSpotX builds on this strategy, proving that the contamination decomposition is non-identifiable from observed counts alone and deriving an anchor-gene constraint that provably restores identifiability. To our knowledge, DeSpotX is the first decontamination method for spatial transcriptomics derived from a formal identifiability analysis, replacing prior-based regularization with a provable guarantee.

## 3 Methods

### 3.1 Problem formulation

Consider a single-cell-resolution spatial transcriptomics dataset of N cells indexed by i∈{1,…,N}. For each cell i, we observe a count vector $x_{i}\in Z_{\geq 0}^{G}$ over G genes, a discrete cluster label $t_{i}\in \{1,\ldots ,T\}$, and a spatial coordinate $s_{i}\in R^{2}$. Let 𝒩(i)⊂{1,…,N} with |𝒩(i)|=K denote the K nearest spatial neighbors of cell i under the Euclidean metric. We model the observed counts as a mixture of the cell’s native transcriptional output and contamination from its local spatial environment:

(1) $$x_{i,g}\sim \text{NB}\left(d_{i}m_{i,g},\;\theta_{g}\right),\;m_{i}=\left(1-\varepsilon_{i}\right)\phi_{t_{i}}\left(z_{i}\right)+\varepsilon_{i}\chi_{i},$$

where $d_{i}=\sum_{g}x_{i,g}$ is the library size, $\theta_{g}\in R_{>0}$ is a gene-wise dispersion parameter, $\phi_{t_{i}}\left(z_{i}\right)\in \Delta^{G-1}$ is the cell’s native expression profile over genes conditioned on its cluster index $t_{i}$ and a latent state $z_{i}\in R^{d_{z}},\chi_{i}\in \Delta^{G-1}$ is the local contamination profile at location $s_{i}$, and $\varepsilon_{i}\in [0,1]$ is the per-cell contamination fraction. We adopt the mean-dispersion parameterization throughout, where NB(μ,θ) denotes the negative binomial with mean μ and variance $\mu +\mu^{2}/\theta$.

The inference task is to recover, for each cell, the native profile $\phi_{t_{i}}\left(z_{i}\right)$, the local contamination profile $\chi_{i}$, and the contamination fraction $\varepsilon_{i}$ from the observation $x_{i}$, the cluster index $t_{i}$, and the spatial context $\left\{\left(x_{j},t_{j},\left\|s_{j}-s_{i}\right\|\right)\;:\;j\in 𝒩(i)\right\}$. The likelihood in Eq. (1) depends on $\left(\varepsilon_{i},\phi_{t_{i}},\chi_{i}\right)$ only through the mixture $m_{i}$, making the decomposition non-identifiable.

**Lemma 1** (Non-identifiability of the native–contamination decomposition). *For any observed mean* $m\in \Delta^{G-1}$, *the set of parameters consistent with* m,

$$𝒮(m)=\left\{(\varepsilon ,\phi ,\chi )\in [0,1]\times \Delta^{G-1}\times \Delta^{G-1}\;:\;(1-\varepsilon )\phi +\varepsilon \chi =m\right\},$$

*contains a continuum of solutions on which the likelihood in* Eq. (1) *is constant*.

A proof is provided in Appendix B.1. Consequently, any decontamination method must impose external constraints on at least one of ϕ,χ, or ε to recover a unique decomposition. DeSpotX imposes such constraints through anchor positions in ϕ and a spatial estimator for χ, and mitigates noise sensitivity through a latent diffusion prior over z.

### 3.2 DeSpotX architecture

**Figure 1:**
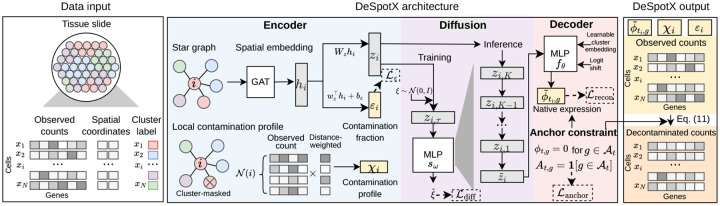
Overview of DeSpotX for decontaminating spatial transcriptomics data.

DeSpotX instantiates Eq. (1) as a deep generative model with four components (Figure 1). A **spatial graph** encoder maps each cell’s spatial context to a latent encoding $z_{i}$ of its native expression state and an estimate of the contamination fraction ${\overset{‾}{\varepsilon }}_{i}$, alongside a local contamination profile $\chi_{i}$ aggregated from cross-cluster spatial neighbors. A **latent diffusion prior** constrains $z_{i}$ to a data-driven distribution of plausible cluster-conditioned latents, preserving biological signal under noisy contamination estimates. A **cluster-conditioned decoder** maps $\left(z_{i},t_{i}\right)$ to the native profile $\phi_{t_{i}}\left(z_{i}\right)$. **Anchor constraints**, inferred automatically from per-cluster expression rates in the data, restore identifiability of the decomposition by fixing $\phi_{t,g}$ to zero at $\left(t,g\right)$ pairs where gene g is not natively expressed in cluster t.

#### Spatial graph encoder.

For each cell i we construct a star graph ${𝒢}_{i}$ in which a single center node, cell i, is connected to K leaf nodes corresponding to the cells in 𝒩(i). Node features are log-transformed counts concatenated with a cluster embedding; edge features are kernel weights $w_{ij}=\text{exp}\left(-\left\|s_{j}-s_{i}\right\|/\rho_{i}\right)$ with adaptive bandwidth $\rho_{i}$ set to the median distance from cell i to its K nearest neighbors. A GATv2 layer [18] aggregates neighbor information into the center, producing a spatial-context embedding $h_{i}\in R^{d_{h}}$. From $h_{i}$, the encoder produces a latent state $z_{i}$ and a contamination-fraction estimate ${\overset{‾}{\varepsilon }}_{i}$:

(2) $$z_{i}=W_{z}h_{i},\;{\overset{‾}{\varepsilon }}_{i}=\sigma \left(w_{\varepsilon }^{⊤}h_{i}+b_{\varepsilon }\right),$$

where $W_{z},w_{\varepsilon }$, and $b_{\varepsilon }$ are learned parameters, and the sigmoid ensures ${\overset{‾}{\varepsilon }}_{i}\in (0,1)$. Implementation details are provided in Appendix C.1.

We estimate the local contamination profile $\chi_{i}$ as a cluster-masked, distance-weighted average over 𝒩(i):

(3) $$\chi_{i}=\frac{1}{Z_{i}}\sum_{j\in 𝒩(i)}w_{ij}\cdot 1\left[t_{j}\neq t_{i}\right]\cdot \frac{x_{j}}{d_{j}},\;Z_{i}=\sum_{j\in 𝒩\left(i\right)}w_{ij}\cdot 1\left[t_{j}\neq t_{i}\right],$$

with fallback to a dataset-level cross-cluster ambient profile when no other-cluster neighbor is available. The mask $1\left[t_{j}\neq t_{i}\right]$ restricts $\chi_{i}$ to a cross-cluster aggregation of the local spatial environment, isolating shared contamination from the center cell’s own native signal. This masking excludes within-cluster contamination; the resulting error in ${\overset{ˆ}{\phi }}_{i}$ is bounded by $\varepsilon_{i}\cdot \sigma_{i}^{\text{within}}$, where $\sigma_{i}^{\text{within}}$ measures within-cluster expression heterogeneity (Appendix B.2, validated in Appendix I).

#### Latent diffusion prior.

The spatial estimator $\chi_{i}$ carries error that propagates into the recovered decomposition. Writing $\chi_{i}=\chi_{i}^{*}+\eta_{i}$ with $E\left[\eta_{i}\right]=0$, solving the mixture equation for $\phi_{t_{i}}$ gives

(4) $${\overset{ˆ}{\phi }}_{t_{i},g}=\phi_{t_{i},g}-\frac{\varepsilon_{i}}{1-\varepsilon_{i}}\eta_{i,g}.$$

The relative distortion $\eta_{i,g}/\phi_{t_{i},g}$ grows large when $\phi_{t_{i},g}$ is small, leading to spurious removal of low-but-real signal. We address this by regularizing z with a diffusion prior over plausible cluster-conditioned latents.

We model the distribution of cluster-conditioned latents via a denoising diffusion process [19]. A score network $s_{\omega }$ is trained alongside the encoder and decoder to predict noise added to encoder latents:

(5) $${ℒ}_{\text{diff}}=E_{\tau ,\xi ,i}{\left\|s_{\omega }\left(\text{sg}\left[z_{i,\tau }\right],\tau ,\text{sg}\left[h_{i}\right],t_{i}\right)-\xi \right\|}_{2}^{2},$$

where $z_{i,\tau }$ is the noised latent at diffusion step τ,ξ is the injected noise, and sg[⋅] denotes stopgradient, which prevents the encoder from collapsing z to a degenerate distribution that $s_{\omega }$ trivially denoises. At inference, $z_{i}$ is refined by a DDIM [20] reverse trajectory, projecting it onto the learned manifold before decoding. Implementation details are provided in Appendix C.2.

#### Cluster-conditioned decoder.

The decoder maps the latent state and cluster index to the native expression profile:

(6) $$\phi_{t_{i}}\left(z_{i}\right)=\text{softmax}\left(f_{\theta }\left(z_{i},e_{t_{i}}\right)+b_{t_{i}}\right),$$

where $f_{\theta }$ is a multilayer perceptron, $e_{t_{i}}$ is a learned cluster embedding, and $b_{t_{i}}\in R^{G}$ is a cluster-specific logit shift initialized from the log-mean expression of cluster $t_{i}$. Implementation details are provided in Appendix C.3.

#### Anchor constraints.

By Lemma 1, identifiability requires external constraints. We introduce *anchor genes*: for each cluster t, let ${𝒜}_{t}\subseteq \{1,\ldots ,G\}$ denote a set of genes not natively expressed in cluster t, and let $A_{t,g}=1\left[g\in {𝒜}_{t}\right]$. The set ${𝒜}_{t}$ is identified from per-cluster expression rates (Appendix C.4).

**Lemma 2** (Identifiability of the decomposition under anchor constraints). *Fix a cell* i *with cluster index* $t_{i}$, *and assume* $\chi_{i}$ *is constructed without using* $x_{i}$. *Suppose* $\phi_{t,g}=0$ *for all* $g\in {𝒜}_{t}$, *and that* $\chi_{i,g}>0$ *for at least one* $g\in {𝒜}_{t_{i}}$. *Then* $\varepsilon_{i}$ *is identifiable from the observations at anchor positions, and* $\phi_{t_{i}}$ *is identifiable on non-anchor positions whenever* $\varepsilon_{i}<1$.

A proof is provided in Appendix B.3.

#### Training objective.

The four components are trained together under

(7) $$ℒ={ℒ}_{\text{recon}}+\lambda_{A}\;{ℒ}_{\text{anchor}}+\lambda_{D}\;{ℒ}_{\text{diff}}+\lambda_{E}\;{ℒ}_{\varepsilon },$$

where the reconstruction loss is the per-cell negative log-likelihood under Eq. (1),

(8) $${ℒ}_{\text{recon}}=-\frac{1}{N}\sum_{i}\sum_{g}\text{log}\;p_{\text{NB}}\left(x_{i,g};d_{i}\cdot m_{i,g},\theta_{g}\right),\;m_{i}=\left(1-{\overset{‾}{\varepsilon }}_{i}\right)\phi_{t_{i}}\left(z_{i}\right)+{\overset{‾}{\varepsilon }}_{i}\chi_{i},$$

the anchor penalty

(9) $${ℒ}_{\text{anchor}}=\frac{1}{N}\sum_{i}\sum_{g}A_{t_{i},g}\cdot \left(1-{\overset{‾}{\varepsilon }}_{i}\right)\phi_{t_{i},g}\left(z_{i}\right)\cdot d_{i}$$

penalizes nonzero $\phi_{t_{i},g}$ at anchor positions, the diffusion loss ${ℒ}_{\text{diff}}$ is given in Eq. (5), and the contamination-fraction regularizer

(10) $${ℒ}_{\varepsilon }=-\frac{1}{N}\sum_{i}\text{log}\;\text{Beta}\left({\overset{‾}{\varepsilon }}_{i};\mu_{\varepsilon }\nu ,\left(1-\mu_{\varepsilon }\right)\nu \right)$$

pulls ${\overset{‾}{\varepsilon }}_{i}$ toward a learnable target $\mu_{\varepsilon }$ with fixed concentration ν. The anchor weight $\lambda_{A}$ is linearly increased from zero over the first $E_{\text{warm}}$ epochs, letting the decoder establish reasonable profiles before the constraint is enforced fully. Optimization details are provided in Appendix C.5.

### 3.3 Inference

With all components fixed after training, denoised counts are produced as follows. The star graph encoder yields the spatial-context embedding $h_{i}$, the latent state $z_{i}$, the contamination fraction ${\overset{‾}{\varepsilon }}_{i}$, and the local contamination profile $\chi_{i}$. DDIM refinement then projects $z_{i}$ onto the learned manifold, and the resulting ${\tilde{z}}_{i}$ is decoded into the refined native profile ${\tilde{\phi }}_{t_{i}}\left({\tilde{z}}_{i}\right)$. Each observation is then reweighted by the native component’s contribution to the mixture:

(11) $${\overset{ˆ}{x}}_{i,g}=x_{i,g}\cdot \frac{\left(1-{\overset{‾}{\varepsilon }}_{i}\right){\tilde{\phi }}_{t_{i},g}}{\left(1-{\overset{‾}{\varepsilon }}_{i}\right){\tilde{\phi }}_{t_{i},g}+{\overset{‾}{\varepsilon }}_{i}\chi_{i,g}}\cdot \left(1-A_{t_{i},g}\right),$$

where the final factor sets anchor positions to zero in the output.

## 4 Experiments

### 4.1 Datasets

We evaluate DeSpotX and baseline methods on five publicly available single-cell-resolution spatial transcriptomics datasets (Table 1). The five datasets span the major imaging- and sequencing-based platforms (Xenium [1], Xenium 5K Prime [21], CosMx [4], MERFISH [3], and Stereo-seq [5]), four tissue contexts (human breast cancer, human non-small cell lung cancer, mouse brain, and mouse embryo), and panel sizes ranging from 313 to 18,582 genes. Cell-cluster annotations are taken from the original studies where available; for Xenium5k_Breast, annotations are obtained by CellTypist [22] label transfer using a published human breast atlas as reference [23]. All datasets are used as published, without additional quality control or filtering; raw integer counts are provided to all methods, with spatial centroids and cell-cluster annotations additionally provided to methods that use them.

### 4.2 Spike-in simulations

Spatial transcriptomics data lack ground truth for ambient contamination, so we construct a spike-in benchmark from real datasets.

#### 4.2.1 Simulation design

To prevent the benchmark from favoring DeSpotX, we sample contamination counts from a Poisson likelihood (rather than DeSpotX’s negative-binomial) and draw per-cell contamination rates from a Beta distribution (rather than DeSpotX’s regularized encoder estimate). For each of the five datasets in Table 1, we sample 40,000 cells and inject synthetic ambient counts at six noise levels $\overset{‾}{\varepsilon }\in \{0,\;5,\;10,\;20,\;30,\;40\}\%$. For each cell, we (i) construct a local ambient profile from its nearest cross-cell-cluster neighbors, (ii) draw a per-cell contamination rate from a Beta distribution with mean $\overset{‾}{\varepsilon }$, and (iii) sample per-gene contamination counts from the resulting per-cell, per-gene Poisson rates. The spiked counts $s_{i,g}=n_{i,g}+c_{i,g}$ are provided to each method as input, while the native counts $n_{i,g}$, the per-gene contamination labels $y_{i,g}=1\left[c_{i,g}>0\right]$, and the realized contamination fraction $\varepsilon_{\text{true}}=\sum_{i,g}c_{i,g}/\sum_{i,g}s_{i,g}$ are retained as ground truth. Details are provided in Appendix D.

#### 4.2.2 Benchmark evaluation

##### Baselines.

We compare DeSpotX against four published decontamination baselines, including SoupX [13], DecontX [12], ResolVI [8], and SpaceBender [10], all run with default settings. Cell-Bender [14] is excluded because it requires empty-droplet observations that are unavailable for the ST platforms, and DenoIST [9] is excluded because it requires per-molecule transcript files that are unavailable in our benchmark. All methods receive the same spiked counts $s_{i,g}$ as input; spatial coordinates and cell-cluster labels are additionally provided to methods that use them. Each method outputs decontaminated counts ${\overset{ˆ}{n}}_{i,g}$.

##### Metrics.

We evaluate each method on three complementary metrics. (i) AUROC scores how well each method’s removed contamination ${\text{adj}}_{i,g}=s_{i,g}-{\overset{ˆ}{n}}_{i,g}$ predicts the binary label $y_{i,g}=1\left[c_{i,g}>0\right]$ of synthetic contamination. (ii) per-cell calibration error (PCE) and (iii) global calibration error (GCE) measure the absolute deviation between each method’s implied contamination fraction and the true rate, computed per cell and dataset-wide respectively. Since spatial transcriptomics data contain platform-derived contamination, both PCE and GCE are computed after subtracting each method’s removal at $\overset{‾}{\varepsilon }=0$.

##### Benchmark performance.

Table 2 reports each metric averaged across the five non-zero noise levels, with DeSpotX achieving the best performance on every metric and every dataset. On AUROC, DeSpotX is uniformly above 0.94, while the next-best method, ResolVI, averages 0.892 across the five datasets. The non-spatial methods, SoupX and DecontX, achieve AUROC scores below 0.74 on every dataset, indicating that contamination in ST data is spatially structured. DeSpotX also achieves the lowest PCE and GCE on every dataset, with the methods ranking similarly on PCE and GCE, indicating that DeSpotX’s global calibration arises from correct per-cell localization rather than averaging out per-cell errors. DeSpotX’s advantage is widest on the largest gene panels (Xenium5k_Breast and Stereo-seq_E14), where its GCE is 1.32 and 3.72 percentage points (pp) respectively, while no other method falls below 8 pp on Xenium5k_Breast or 13 pp on Stereo-seq_E14.

##### Ablation study.

We assess each architectural component by removing it from the full model on Xenium_Breast and MERFISH_Brain (full results in Appendix E). Removing the identifiability constraints produces the largest degradation, with GCE rising from 10 to over 70 pp on Xenium_Breast and from 5 to 17 pp on MERFISH_Brain. Removing the spatial graph encoder, the cluster mask, or the diffusion prior each reduces performance on all three metrics, confirming that every component contributes to DeSpotX’s accuracy. Beyond aggregate metrics, the diffusion prior specifically protects low-expression marker genes from over-correction (full results in Appendix F).

##### Robustness and consistency.

DeSpotX is robust to the construction of the anchor mask, the perturbation of the cell-cluster annotation, the number of spatial neighbors K, and the cell-cluster annotation method (Appendix G). The performance of DeSpotX is consistent across contamination levels, and its margin over baselines grows at higher contamination levels (Appendix H). The within-cluster contamination bound is validated on spike-in simulations (Appendix I).

### 4.3 Application to real spatial transcriptomics data

We apply DeSpotX to the full five datasets in Table 1; it runs much faster than other ST methods (Appendix J). We then assess whether decontamination improves downstream analyses.

#### 4.3.1 Cell-cluster identity

**Figure 2:**
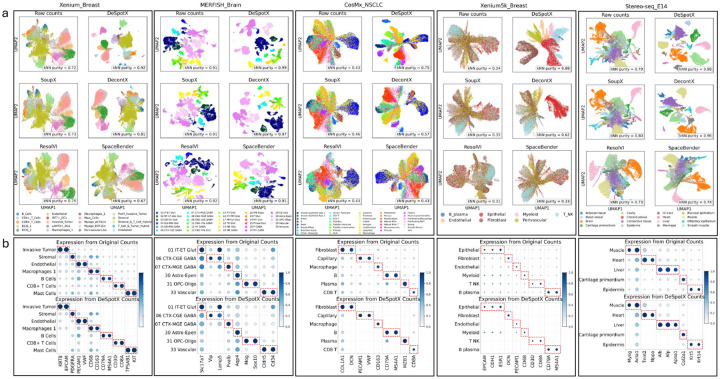
DeSpotX yields better-separated cluster structure and improved marker-gene specificity on real spatial transcriptomics data. (a) UMAP embeddings of raw counts and decontaminated counts from each method, across the five datasets, colored by cell cluster. kNN purity is shown in each panel. (b) Marker-gene dotplots for raw and DeSpotX-decontaminated counts across cell clusters in each dataset; red boxes mark canonical marker-cluster pairs.

We first examine whether decontamination produces clearer separation of cell clusters and tighter localization of marker-gene expression to canonical clusters. Figure 2a shows UMAP embeddings of the raw counts and the decontaminated counts from DeSpotX and the four baselines across the five datasets. Each panel reports the kNN purity at k=20, defined as the fraction of each cell’s k nearest neighbors in UMAP space that share its cluster label; higher values indicate cleaner cluster separation. DeSpotX yields the highest kNN purity on every dataset and produces visibly better-separated clusters than the raw counts, while the baselines yield embeddings with less clear cluster separation.

Figure 2b compares marker-gene dotplots for the raw counts and the DeSpotX-decontaminated counts across cell clusters in each dataset. After decontamination, expression of canonical markers is reduced in non-expressing clusters while remaining concentrated in their expected clusters (red boxes). This pattern is consistent with the removal of ambient contamination from non-expressing cells, with native expression preserved in expressing cells. The same comparison for the four baseline methods is provided in Appendix K.

#### 4.3.2 Spatial coherence

**Figure 3:**
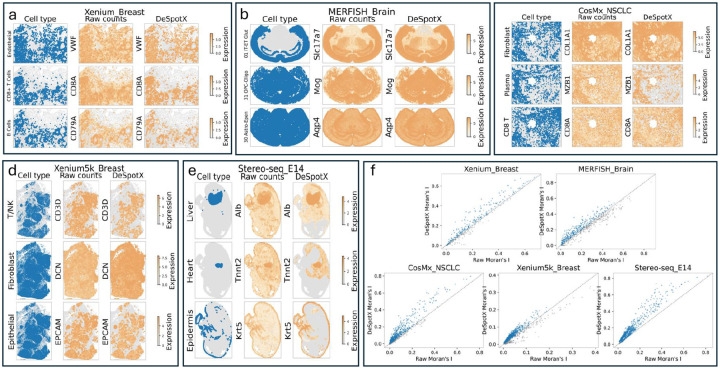
DeSpotX-decontaminated expression is spatially coherent. (a-e) Spatial maps of canonical cell-cluster markers in each dataset, comparing raw and DeSpotX-decontaminated counts. (f) Pergene Moran’s I for raw and DeSpotX-decontaminated counts across the five datasets.

We next assess whether DeSpotX decontamination preserves the spatial structure of marker expression. Figure 3a–e shows the spatial distribution of canonical cell-cluster markers in the raw and DeSpotX-decontaminated counts. In the raw counts, marker expression is detected throughout the tissue, including in regions where the canonical expressing cluster is absent. After DeSpotX, marker expression is concentrated within the canonical clusters and the surrounding tissue is largely cleared, indicating that DeSpotX removes ambient signal in non-expressing regions while preserving expression in regions where the marker is natively transcribed. Spatial maps of the removed counts further show that DeSpotX’s removal is spatially structured, concentrating near canonical expressing clusters, consistent with the assumption that contamination spreads locally from source cells (Appendix L).

Figure 3f reports per-gene Moran’s I for the raw and DeSpotX-decontaminated counts across the five datasets. Moran’s I measures whether gene expression is spatially autocorrelated, with higher values indicating that cells with similar expression are spatially close. Most genes show higher Moran’s I after DeSpotX, with the largest gains in genes that already exhibit some spatial structure in the raw data, indicating that decontamination preserves and strengthens biologically meaningful spatial patterns. The same comparison for baseline methods is provided in Appendix M.

#### 4.3.3 Iterative decontamination and cell-cluster annotation

**Figure 4:**
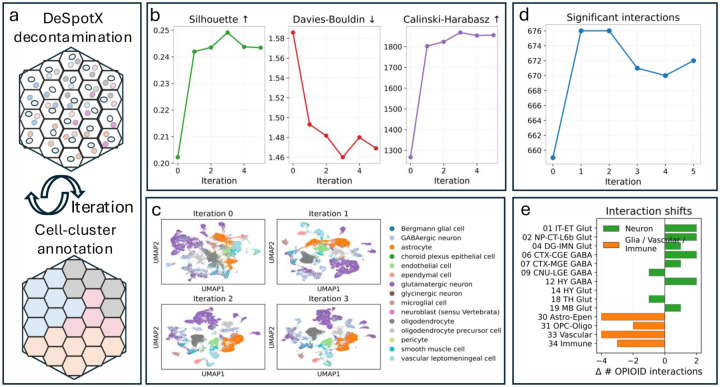
Iterative DeSpotX decontamination and cell-cluster annotation on MERFISH_Brain. (a) Schematic of the iterative procedure. (b) Cluster metrics across iterations. (c) UMAP embeddings of the decontaminated counts at iterations 0–3. (d) Total number of significant cell-cell interactions inferred by CellChat across iterations. (e) Change in the number of significant OPIOID-pathway interactions per sender cell-cluster between iteration 0 and iteration 5.

DeSpotX requires a cell-cluster annotation as input, but the quality of this annotation depends on the data, which itself contains contamination. We therefore evaluate whether iterating between decontamination and cell-cluster annotation produces progressively better results. At each iteration, DeSpotX decontaminates the raw counts using the previous iteration’s annotation, and the decontaminated counts are re-annotated by a CellTypist classifier [22] trained on a reference atlas [24] (Figure 4a).

We apply this procedure to MERFISH_Brain for five iterations. Cluster-validity metrics improve and stabilize by iteration 3 (Figure 4b), with Silhouette rising from 0.20 to 0.24, Davies-Bouldin falling from 1.59 to 1.47, and Calinski-Harabasz increasing from 1,267 to 1,856. The UMAP embeddings show progressively cleaner separation of the brain cell types (Figure 4c). We run CellChat [25] on inferred ligand-receptor signaling networks at each iteration. The number of significant interactions stabilizes by iteration 2–3 (Figure 4d). Within the OPIOID pathway, the sender distribution shifts (Figure 4e), moving away from glial, vascular, and immune senders toward neuronal populations, consistent with the neuronal origin of opioid peptides [26, 24]. The same iterative procedure applied to Xenium_Breast also shows improvements in downstream analyses (Appendix N).

## 5 Discussion

We have presented DeSpotX, the first decontamination method for spatial transcriptomics derived from a formal identifiability analysis. Whereas existing methods rely on heuristic priors, we prove that the contamination decomposition is non-identifiable from observed counts alone and that anchor genes provably restore identifiability. DeSpotX outperforms existing methods on every benchmarked dataset and produces more biologically coherent outputs on real tissue, with iteration between decontamination and cell-cluster annotation further refining these outcomes.

We outline two limitations along with corresponding future work. First, DeSpotX is designed for single-cell-resolution ST platforms; spot-resolution platforms such as Visium or Slide-seq aggregate multiple cells per spot, and extending DeSpotX would require modeling cell-cluster mixtures. Second, the identifiability guarantee depends on the anchor genes within each cluster, which may be sparse in highly homogeneous tissues; future work could explore softer anchor formulations.

## Supplementary Material

1

## Figures and Tables

**Table 1:** Single-cell-resolution spatial transcriptomics datasets.

Dataset name	Technology	Tissue	# cells	# genes	# cell clusters
Xenium_Breast [1]	Xenium	Breast cancer	118,370	313	20
MERFISH_Brain [3]	MERFISH	Mouse brain	115,779	550	29
CosMx_NSCLC [4]	CosMx	Non-small cell lung cancer	99,185	980	38
Xenium5k_Breast [21]	Xenium 5K Prime	Breast cancer	577,258	5,101	7
Stereo-seq_E14 [5]	Stereo-seq	Mouse embryo (E14.5)	92,928	18,582	16

**Table 2:** Benchmark comparison across five spatial transcriptomics datasets. We report AUROC, per-cell calibration error (PCE, in percentage points), and global calibration error (GCE, in percentage points). Values are mean ± std across the non-zero noise levels. Best value per row in bold.

Dataset	Metric	DeSpotX	SoupX [13]	DecontX [12]	ResolVI [8]	SpaceBender [10]
Xenium_Breast	AUROC↑	**0.955 ± 0.007**	0.606 ± 0.069	0.677 ± 0.014	0.851 ± 0.002	0.805 ± 0.009
	PCE↓	**15.79 ± 10.39**	16.45 ± 9.87	18.35 ± 12.48	20.62 ± 14.27	16.33 ± 9.58
	GCE↓	**10.03 ± 4.78**	11.26 ± 4.60	13.11 ± 6.84	15.71 ± 8.76	10.21 ± 6.04
MERFISH_Brain	AUROC↑	**0.947 ± 0.008**	0.631 ± 0.008	0.721 ± 0.013	0.812 ± 0.009	0.824 ± 0.013
	PCE↓	**10.83 ± 7.14**	20.66 ± 13.94	16.99 ± 11.75	17.60 ± 13.58	12.72 ± 7.79
	GCE↓	**5.27 ± 1.92**	16.08 ± 8.41	12.97 ± 6.87	12.19 ± 7.98	7.57 ± 3.68
CosMx_NSCLC	AUROC↑	**0.967 ± 0.003**	0.696 ± 0.011	0.683 ± 0.002	0.886 ± 0.005	0.700 ± 0.013
	PCE↓	**13.64 ± 9.22**	15.43 ± 11.55	20.13 ± 13.45	21.15 ± 12.76	16.09 ± 9.87
	GCE↓	**7.64 ± 3.90**	9.31 ± 6.37	14.52 ± 7.57	15.61 ± 7.34	12.61 ± 6.08
Xenium5k_Breast	AUROC↑	**0.997 ± 0.000**	0.705 ± 0.024	0.732 ± 0.006	0.980 ± 0.000	0.604 ± 0.020
	PCE↓	**8.06 ± 4.39**	16.16 ± 10.61	21.74 ± 14.30	19.07 ± 13.79	13.54 ± 7.38
	GCE↓	**1.32 ± 1.15**	8.09 ± 5.16	15.57 ± 8.20	13.15 ± 7.94	10.74 ± 5.16
Stereo-seq_E14	AUROC↑	**0.984 ± 0.001**	0.719 ± 0.051	0.721 ± 0.002	0.929 ± 0.004	0.904 ± 0.010
	PCE↓	**15.74 ± 8.71**	22.49 ± 17.03	19.78 ± 13.58	21.12 ± 13.91	18.86 ± 11.81
	GCE↓	**3.72 ± 1.93**	17.87 ± 11.44	15.11 ± 8.05	16.51 ± 8.36	13.71 ± 6.54
