# Supplementary material for: DeSpotX: Identifiability-Based Decontamination for Spatial Transcriptomics
Source: bioRxiv. 2026 May 14:2026.05.12.724704. Preprint. [Version 1] doi: 10.64898/2026.05.12.724704 (PMC13192984; doi:10.64898/2026.05.12.724704)
Supplement: 1 [file NIHPP2026.05.12.724704V1-supplement-1.pdf]

bioRxiv preprint doi: https://doi.org/10.64898/2026.05.12.724704; this version posted May 14, 2026. The copyright holder for this preprint (which was not certified by peer review) is the author/funder, who has granted bioRxiv a license to display the preprint in perpetuity. It is made available under a CC-BY 4.0 International license.

A Comparison of decontamination methods

Table 3 summarizes the key differences between DeSpotX and existing decontamination methods for scRNA-seq and ST data. The methods differ in the data type, the model architecture, the contamination estimator, the use of cluster annotations, and the constraint used to ensure identifiability. DeSpotX is the only method that introduces an explicit identifiability constraint through anchor genes, isolating spatial contamination from native expression in a cluster-aware framework.

B Proofs

B.1 Proof of Lemma 1

We want to show two things: (i) the set $\mathcal{S}(m)$ contains infinitely many triples, and (ii) the likelihood in Eq. (1) is the same at all of them.

bioRxiv preprint doi: https://doi.org/10.64898/2026.05.12.724704; this version posted May 14, 2026. The copyright holder for this preprint (which was not certified by peer review) is the author/funder, who has granted bioRxiv a license to display the preprint in perpetuity. It is made available under a CC-BY 4.0 International license.

Table 3: Comparison of decontamination methods for scRNA-seq and ST data.

Method	Data type	Architecture	Contamination estimator	Cluster-aware	Identifiability constraint
SoupX [13]	scRNA-seq	Linear subtraction	Global ambient profile from empty droplets	No	None
DecontX [12]	scRNA-seq	Bayesian mixture	Global ambient profile, cluster-aggregated	Yes	None
CellBender [14]	scRNA-seq	Deep generative model	Learned ambient prior from empty droplets	No	None
SpaceBender [10]	ST	Deep generative model	Local ambient profile from spatial neighbors	No	None
DenoIST [9]	ST	Mixture model	Local Poisson mixture per cell	No	None
ResolVI [8]	ST	Deep generative model	Latent decomposition with neighbor diffusion	No	None
**DeSpotX**	**ST**	**Diffusion-regularized generative model**	**Cluster-isolated spatial contamination**	**Yes**	**Anchor genes**

Fix $m \in \Delta^{G-1}$. For any $\varepsilon \in [0, 1)$ and any $\chi \in \Delta^{G-1}$ satisfying $\varepsilon\chi_g \le m_g$ for all g, define

$$\phi = \frac{m - \varepsilon\chi}{1 - \varepsilon}.$$

We check that the triple $(\varepsilon, \phi, \chi)$ lies in $\mathcal{S}(m)$, i.e., that it satisfies the conditions in the Lemma's definition. First, ϕ is a valid probability vector; each coordinate is non-negative because $\varepsilon\chi_g \le m_g$, and the coordinates sum to one,

$$\sum_g \phi_g = \frac{\sum_g m_g - \varepsilon \sum_g \chi_g}{1 - \varepsilon} = \frac{1 - \varepsilon}{1 - \varepsilon} = 1,$$

using $\sum_g m_g = \sum_g \chi_g = 1$.

Second, ϕ satisfies the mixture equation: substituting the definition of ϕ gives

$$(1 - \varepsilon)\phi + \varepsilon\chi = (1 - \varepsilon) \cdot \frac{m - \varepsilon\chi}{1 - \varepsilon} + \varepsilon\chi = m - \varepsilon\chi + \varepsilon\chi = m.$$

Hence $(\varepsilon, \phi, \chi) \in \mathcal{S}(m)$.

Since ε and χ can be chosen from a continuum of admissible values, and each choice produces a distinct triple, $\mathcal{S}(m)$ is a continuum, proving (i). For (ii), the likelihood in Eq. (1) depends on $(\varepsilon, \phi, \chi)$ only through the mixture m, so it is constant across $\mathcal{S}(m)$.

B.2 Error under within-cluster contamination

DeSpotX's cross-cluster mask (Eq. (3)) excludes same-cluster neighbors when estimating contamination, leaving a within-cluster residual in $\hat{\phi}_i$. We show this residual contributes a native-profile error bounded by the contamination level times the within-cluster heterogeneity, while $\hat{\varepsilon}_i$ recovers the cross-cluster contamination rate.

Decompose the true contamination profile at cell i as

$$\chi_i^* = \alpha_i \chi_i^{\text{cross}} + (1 - \alpha_i) \chi_i^{\text{within}}, \qquad \alpha_i \in [0, 1], \tag{12}$$

where α_i is the cross-cluster fraction. Substituting into Eq. (1) gives

$$m_i = (1 - \varepsilon_i^*) \phi_{t_i}(z_i) + \varepsilon_i^* \alpha_i \chi_i^{\text{cross}} + \varepsilon_i^*(1 - \alpha_i) \chi_i^{\text{within}}, \tag{13}$$

while DeSpotX fits

$$m_i = (1 - \hat{\varepsilon}_i) \hat{\phi}_i + \hat{\varepsilon}_i \chi_i^{\text{cross}}. \tag{14}$$

Let

$$\sigma_i^{\text{within}} = \left\| \chi_i^{\text{within}} - \phi_{t_i}(z_i) \right\| \tag{15}$$

denote the within-cluster heterogeneity at cell i, the deviation between cell i's native expression and the average expression of its same-cluster spatial neighbors.

bioRxiv preprint doi: https://doi.org/10.64898/2026.05.12.724704; this version posted May 14, 2026. The copyright holder for this preprint (which was not certified by peer review) is the author/funder, who has granted bioRxiv a license to display the preprint in perpetuity. It is made available under a CC-BY 4.0 International license.

Lemma 3 (Error from within-cluster contamination)**.** *Under the conditions of Lemma 2, with $\varepsilon_i^* < 1$, define the scaling factor*

$$\beta_i \;=\; \frac{\varepsilon_i^*(1-\alpha_i)}{1-\alpha_i\varepsilon_i^*}. \tag{16}$$

The unique fit of Eq. (14) *to the true mixture in Eq.* (13) *satisfies $\hat{\varepsilon}_i = \alpha_i\varepsilon_i^*$ and*

$$\left\|\hat{\phi}_i - \phi_{t_i}(z_i)\right\| \;=\; \beta_i \cdot \sigma_i^{\text{within}} \;\le\; \varepsilon_i^* \cdot \sigma_i^{\text{within}}. \tag{17}$$

Proof. Equating Eqs. (13) and (14) gives

$$(1-\hat{\varepsilon}_i)\hat{\phi}_i + \hat{\varepsilon}_i\chi_i^{\text{cross}} \;=\; (1-\varepsilon_i^*)\phi_{t_i}(z_i) + \varepsilon_i^*\alpha_i\chi_i^{\text{cross}} + \varepsilon_i^*(1-\alpha_i)\chi_i^{\text{within}}.$$

At any anchor position $g \in \mathcal{A}_{t_i}$, $\phi_{t_i,g}(z_i) = 0$ by definition. Since χ_i^{within} averages expression from cluster-t_i cells, which are zero at anchor positions, $\chi_{i,g}^{\text{within}} = 0$ as well. The anchor constraint forces $\hat{\phi}_{i,g} = 0$, so the equation at g reduces to $\hat{\varepsilon}_i\chi_{i,g}^{\text{cross}} = \varepsilon_i^*\alpha_i\chi_{i,g}^{\text{cross}}$. Since at least one anchor satisfies $\chi_{i,g}^{\text{cross}} > 0$, $\hat{\varepsilon}_i = \alpha_i\varepsilon_i^*$. Substituting back,

$$(1-\alpha_i\varepsilon_i^*)\hat{\phi}_i \;=\; (1-\varepsilon_i^*)\phi_{t_i}(z_i) + \varepsilon_i^*(1-\alpha_i)\chi_i^{\text{within}}.$$

Dividing by $1-\alpha_i\varepsilon_i^*$ and subtracting $\phi_{t_i}(z_i)$ gives $\hat{\phi}_i - \phi_{t_i}(z_i) = \beta_i[\chi_i^{\text{within}} - \phi_{t_i}(z_i)]$, and Eq. (17) follows by taking norms.

Remarks. The error in Eq. (17) is the product of two small quantities. The scaling factor β_i is bounded by ε_i^* (for example, $\beta_i = 0.07$ at $\varepsilon_i^* = 0.2$ and $\alpha_i = 0.7$). The within-cluster heterogeneity σ_i^{within} is small for well-defined clusters.

The recovered contamination fraction $\hat{\varepsilon}_i = \alpha_i\varepsilon_i^*$ is the cross-cluster rate, which drives the downstream artifacts that motivate decontamination, such as cell-type misannotation, smeared marker maps, and spurious cell-cell communication. The within-cluster portion absorbed into $\hat{\phi}_i$ does not cause these artifacts, since the recipient cell expresses similar genes natively.

We defer validation of Lemma 3 on spike-in simulations to Appendix I.

B.3 Proof of Lemma 2

We show two things: (i) ε_i is identifiable from observations at anchor positions, and (ii) ϕ_{t_i} is identifiable at all non-anchor positions. Both follow under the preconditions of the Lemma: χ_i given as input, constructed without using x_i, and at least one anchor with $\chi_{i,g} > 0$.

Recall that $m_i = (1-\varepsilon_i)\phi_{t_i} + \varepsilon_i\chi_i$ denotes the mixture mean satisfying $\mathbb{E}[x_i/d_i] = m_i$. Because χ_i is not a function of x_i, the identification of ε_i and ϕ_{t_i} from x_i is not circular.

For (i), consider any anchor position $g \in \mathcal{A}_{t_i}$. By the anchor assumption $\phi_{t_i,g} = 0$, the mixture equation reduces to

$$m_{i,g} = \varepsilon_i\chi_{i,g}.$$

Since by assumption there exists $g \in \mathcal{A}_{t_i}$ with $\chi_{i,g} > 0$, solving gives

$$\varepsilon_i = \frac{m_{i,g}}{\chi_{i,g}},$$

which is a function of observable quantities and the (independently obtained) χ_i.

For (ii), fix ε_i and χ_i as above. For any non-anchor position $g \notin \mathcal{A}_{t_i}$, the mixture equation gives

$$\phi_{t_i,g} = \frac{m_{i,g} - \varepsilon_i\chi_{i,g}}{1-\varepsilon_i},$$

which expresses $\phi_{t_i,g}$ in terms of observables and parameters identified in (i).

Hence both ε_i and ϕ_{t_i} are identifiable.

bioRxiv preprint doi: https://doi.org/10.64898/2026.05.12.724704; this version posted May 14, 2026. The copyright holder for this preprint (which was not certified by peer review) is the author/funder, who has granted bioRxiv a license to display the preprint in perpetuity. It is made available under a CC-BY 4.0 International license.

C Model details

C.1 Spatial graph encoder: implementation details

Node features. For each node j in the star graph $\mathcal{G}_i$, the input feature is

$$\mathbf{n}_j = \left[\ \log(1 + x_j) \,\|\, \mathbf{e}_{t_j}\ \right] \in \mathbb{R}^{G+d_e},$$

where $\log(1+\cdot)$ is applied elementwise to the count vector, $\mathbf{e}_{t_j} \in \mathbb{R}^{d_e}$ is a learned cluster embedding, and $\|$ denotes concatenation. The cluster embedding is initialized randomly and trained jointly with the rest of the model. A linear projection $W_{\text{in}} : \mathbb{R}^{G+d_e} \to \mathbb{R}^{d_h}$ maps $\mathbf{n}_j$ to a hidden representation $\mathbf{n}_j^{(0)} = W_{\text{in}}\mathbf{n}_j$ used as input to the GAT layer.

Edge features. The edge from leaf j to center i carries the kernel weight $w_{ij} = \exp(-\|s_j - s_i\|/\rho_i) \in (0, 1]$ as a single scalar feature, with ρ_i set to the median Euclidean distance from cell i to its K nearest neighbors. The exponential form approximates the spatial decay of free ambient RNA, and the per-cell adaptive bandwidth ρ_i accommodates variation in cell density across the dataset.

GATv2 attention. GATv2 [18] computes attention coefficients of the form

$$\alpha_{ji} \ \propto\ \exp\Big(\mathbf{a}^\top \text{LeakyReLU}\big(W_q\mathbf{n}_i^{(0)} + W_k\mathbf{n}_j^{(0)} + W_e w_{ji}\big)\Big),$$

where W_q, W_k are query and key projections, W_e embeds the scalar edge weight w_{ji}, and $\mathbf{a}$ is a learned scoring vector. We use H attention heads with averaging across heads and per-head output dimension d_h, so the output dimension is d_h. For batched training, B star graphs are concatenated into a single disjoint graph and the center nodes are recovered by index after the forward pass.

Readouts from h_i. Two linear heads applied to the center's spatial-context embedding h_i produce the latent state and the contamination-fraction estimate in Eq. (2):

$$z_i = W_z h_i, \tag{18}$$

$$\bar{\varepsilon}_i = \sigma\big(w_\varepsilon^\top h_i + b_\varepsilon\big), \tag{19}$$

where W_z, w_ε, and b_ε are learned parameters. We initialize $b_\varepsilon = \text{logit}(0.20)$.

Hyperparameters. Default values used in our experiments: GAT hidden dimension $d_h = 256$, $H = 4$ attention heads, cluster embedding dimension $d_e = 32$, $K = 12$ spatial neighbors, and dropout 0.1.

C.2 Latent diffusion prior: implementation details

Forward diffusion. Following the standard DDPM formulation [19], we define a discrete-time forward diffusion on z with T steps and a fixed variance schedule $\{\beta_\tau\}_{\tau=1}^T$. The marginal at step τ is

$$z_\tau \ = \ \sqrt{\bar{\alpha}_\tau}\, z + \sqrt{1 - \bar{\alpha}_\tau}\, \xi, \qquad \bar{\alpha}_\tau = \prod_{s \le \tau}(1 - \beta_s), \qquad \xi \sim \mathcal{N}(0, I).$$

Score network. The score network s_ω is a feedforward MLP whose inputs are the noised latent $z_{i,\tau}$, a sinusoidal embedding of the diffusion step τ, the spatial-context embedding h_i, and a learned cluster embedding indexed by t_i. Its output is a prediction of the noise ξ added at step τ.

Stop-gradients. The stop-gradient operator on h_i and $z_{i,\tau}$ in Eq. (5) blocks diffusion-loss gradients from reaching the encoder, so the encoder is shaped only by the reconstruction loss. This prevents a degenerate solution in which the encoder collapses z_i into a distribution that s_ω trivially denoises, leaving the prior uninformative. It also ensures that the prior tracks the encoder's reconstruction-driven distribution, so refinement at inference projects $\tilde{z}_i$ toward the same distribution the decoder was trained on.

Inference-time refinement. At inference, we noise the encoder output z_i to diffusion step $\tau_{\text{start}} = \lfloor \lambda_{\text{refine}} \cdot T \rfloor$ and run K_{refine} DDIM reverse steps [20] to obtain a refined latent $\tilde{z}_i$, which is decoded to yield $\tilde{\phi}_{t_i}(\tilde{z}_i)$.

bioRxiv preprint doi: https://doi.org/10.64898/2026.05.12.724704; this version posted May 14, 2026. The copyright holder for this preprint (which was not certified by peer review) is the author/funder, who has granted bioRxiv a license to display the preprint in perpetuity. It is made available under a CC-BY 4.0 International license.

Hyperparameters. The score network is a 3-layer MLP with hidden dimension 256 and SiLU activation. The forward diffusion uses $T = 100$ steps with linear schedule β_τ from 10^{-4} to 0.02. At inference, we use noise fraction $\lambda_{\text{refine}} = 0.2$ and $K_{\text{refine}} = 10$ DDIM steps.

C.3 Cluster-conditioned decoder: implementation details

Architecture. The function f_θ in Eq. (6) is a two-layer MLP with hidden dimension d_{dec} and Softplus activations:

$$f_\theta(z_i, e_{t_i}) \;=\; W_2\,\text{Softplus}\big(W_1[z_i \,\|\, e_{t_i}] + b_1\big) + b_2,$$

where $[\,\cdot \,\|\, \cdot\,]$ denotes concatenation, $W_1 \in \mathbb{R}^{d_{\text{dec}} \times (d_z + d_e)}$, $W_2 \in \mathbb{R}^{G \times d_{\text{dec}}}$, $b_1 \in \mathbb{R}^{d_{\text{dec}}}$, $b_2 \in \mathbb{R}^G$, and $e_{t_i} \in \mathbb{R}^{d_e}$ is a learned cluster embedding. We initialize W_2 with Xavier-uniform values scaled by 0.1 and $b_2 = 0$.

Cluster-specific logit shift. The vector $b_{t_i} \in \mathbb{R}^G$ in Eq. (6) is initialized to $b_{t_i,g} = \log \bar{x}_{t_i,g} - \frac{1}{G}\sum_{g'} \log \bar{x}_{t_i,g'}$, where $\bar{x}_{t_i,g}$ is the mean count of gene g over cluster-t_i training cells. After initialization, b_{t_i} is fixed; cluster-specific learnable signal is carried by e_{t_i} and f_θ.

Hyperparameters. Default values used in our experiments: $d_{\text{dec}} = 256$, $d_e = 32$.

C.4 Anchor mask construction: implementation details

Per-cluster expression statistics. Let $r_{t,g}$ denote the fraction of cluster-t cells with nonzero counts for gene g in the training data. We construct the binary anchor mask

$$A_{t,g} \;=\; \mathbf{1}\big[r_{t,g} < \tau_g\big], \tag{20}$$

with adaptive per-gene threshold

$$\tau_g \;=\; \text{clip}\big(\kappa \cdot \max_t r_{t,g},\; \tau_{\min},\; \tau_{\max}\big),$$

which scales with each gene's maximum cluster-level expression rate. Default value used in our experiments: $\kappa = 0.3$.

C.5 Training: implementation details

Loss weights. Default values used in our experiments: $\lambda_A = 50$, $\lambda_D = 1$, $\lambda_E = 1$. Although λ_A appears large relative to λ_D and λ_E, the anchor penalty vanishes once $\phi_{t_i,g}$ is driven to zero at anchor positions, so λ_A only governs the convergence speed of this collapse.

Regularizer on $\bar{\varepsilon}_i$. The Beta-density form in Eq. (10) provides a smooth, bounded penalty whose minimum lies at μ_ε. We use fixed concentration $\nu = 20$ and treat μ_ε as a single global scalar, parameterized by its logit and initialized at $\text{logit}(0.20)$.

Anchor warm-up. The anchor weight λ_A is linearly increased from 0 to 50 over the first $E_{\text{warm}} = 3$ epochs and held at 50 thereafter.

Optimizer. All parameters, including the encoder, decoder, score network, gene-wise dispersion θ, and μ_ε, are optimized with Adam ($\beta_1 = 0.9$, $\beta_2 = 0.999$). We use learning rate 10^{-3}, batch size 128, and gradient clipping at norm 10. Training runs for 10 epochs.

Compute. All experiments were run on a single NVIDIA Tesla T4 GPU (16 GB), with training taking 5–20 minutes per dataset.

D Spike-in simulation details

Ambient profile construction. For each cell i at spatial coordinate $\mathbf{s}_i$, with native counts $\mathbf{n}_i \in \mathbb{N}^G$, library size $d_i = \sum_g n_{i,g}$, and cell-cluster label t_i, we construct a per-cell ambient profile

$$\mathbf{a}_i = \frac{1}{Z_i} \sum_{j \in \mathcal{N}_K^{\neq}(i)} w_{ij} \cdot \frac{\mathbf{n}_j}{d_j}, \qquad w_{ij} = \frac{1}{\|\mathbf{s}_i - \mathbf{s}_j\| + \epsilon_0}, \tag{21}$$

bioRxiv preprint doi: https://doi.org/10.64898/2026.05.12.724704; this version posted May 14, 2026. The copyright holder for this preprint (which was not certified by peer review) is the author/funder, who has granted bioRxiv a license to display the preprint in perpetuity. It is made available under a CC-BY 4.0 International license.

where $\mathcal{N}_K^{\neq}(i)$ is the set of $K = 12$ Euclidean nearest neighbors of cell i restricted to cells with $t_j \neq t_i$, falling back to the unrestricted neighbor set when no other-cluster neighbor exists; $\epsilon_0 = 10^{-6}$ is a numerical stabilizer; and $Z_i = \sum_{j \in \mathcal{N}_K^{\neq}(i)} w_{ij}$ normalizes $\mathbf{a}_i$ to the simplex.

Per-cell contamination rates. We draw per-cell contamination rates from a Beta distribution centered at the noise level $\bar{\varepsilon}$,

$$\varepsilon_i \sim \mathrm{Beta}\big(4,\, 4(1-\bar{\varepsilon})/\bar{\varepsilon}\big), \qquad \mathbb{E}[\varepsilon_i] = \bar{\varepsilon}, \tag{22}$$

with shape parameter fixed at 4 to give moderate per-cell variance.

Spike-in count sampling. Synthetic contamination counts are sampled independently per gene as

$$c_{i,g} \sim \mathrm{Poisson}\big(\varepsilon_i\, d_i\, a_{i,g}\big), \tag{23}$$

and the spiked counts delivered to each method are $s_{i,g} = n_{i,g} + c_{i,g}$.

E Ablation studies

We perform ablation studies on `Xenium_Breast` and `MERFISH_Brain` to assess the contribution of each architectural component. We compare the full DeSpotX model against four ablations, each of which removes one component while keeping the rest of the architecture intact.

Ablation conditions. Each ablation removes a single component from the full model.

- *w/o spatial*: spatial information is removed. The GAT layer is replaced by an MLP applied to each cell's own counts, and χ_i is replaced by a single dataset-level ambient profile shared across cells.
- *w/o cluster-mask*: cluster conditioning is removed. The cross-cluster mask in χ_i (Eq. 3) is dropped so all neighbors contribute regardless of cluster identity, and the cluster-specific decoder bias b_{t_i} is no longer warm-started from per-cluster expression statistics.
- *w/o diffusion*: the latent diffusion prior is disabled. The diffusion loss term is set to zero during training (setting $\lambda_D = 0$), and the DDIM refinement step is skipped at inference, so the encoder latent z_i is decoded directly.
- *w/o identifiability*: identifiability constraints are removed. This includes the anchor penalty (setting $\lambda_A = 0$), the Beta prior on $\bar{\varepsilon}_i$ (setting $\lambda_E = 0$), and the architectural priors that contribute to identifiability. With these constraints removed, the negative-binomial likelihood and the simplex constraint on ϕ remain, corresponding to the unconstrained setting of Lemma 1.

Results. Figure 5 shows that the full model achieves the best mean performance on every metric and dataset, indicating that each architectural component contributes to DeSpotX's overall performance. The largest effect comes from removing the identifiability constraints. On `Xenium_Breast`, PCE rises from a median of 16 to 60 pp and GCE rises from 10 to above 70 pp, with both metrics also showing greatly increased variance across noise levels. AUROC drops substantially and becomes highly variable. On `MERFISH_Brain`, the same ablation raises PCE from a median of 10 to 20 pp and GCE from 5 to 17 pp, with AUROC dropping to a median of <0.90. These results are consistent with Lemma 1, which states that without external constraints, the $(\phi, \varepsilon, \chi)$ decomposition is not uniquely determined.

Removing the spatial information produces consistent degradation across all three metrics on both datasets, confirming that the spatial graph encoder and the local contamination estimator are central to DeSpotX's accuracy. Removing the cluster-mask and the diffusion prior also degrades all three metrics on both datasets, with smaller effects than the spatial ablation. The cluster-mask supplies cell-cluster information that helps separate native from contaminating signal, whereas the diffusion prior reduces distortions introduced by the contamination estimator; the results confirm that both components contribute to DeSpotX's performance.

bioRxiv preprint doi: https://doi.org/10.64898/2026.05.12.724704; this version posted May 14, 2026. The copyright holder for this preprint (which was not certified by peer review) is the author/funder, who has granted bioRxiv a license to display the preprint in perpetuity. It is made available under a CC-BY 4.0 International license.

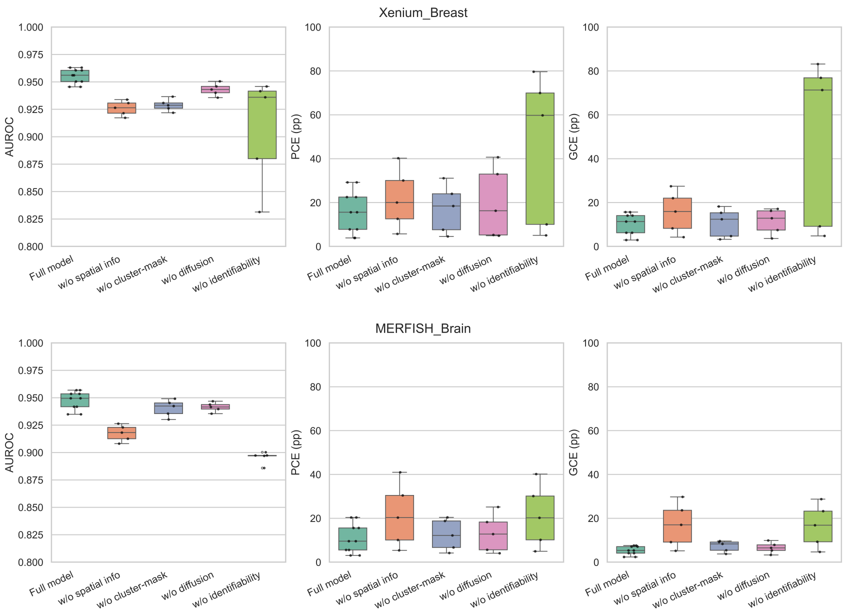

Figure 5: Ablation studies on `Xenium_Breast` (top row) and `MERFISH_Brain` (bottom row), reporting AUROC, PCE, and GCE across the five non-zero noise levels. Each box summarizes the distribution of values across noise levels. Higher AUROC is better; PCE and GCE are reported in percentage points and lower is better.

F Effect of the diffusion prior on low-but-real expression

We test whether DeSpotX's diffusion prior helps preserve low-but-real expression of established marker genes. We use the spike-in benchmark data at $\bar{\varepsilon} = 0$ (no injected contamination) on `Xenium_Breast` and `MERFISH_Brain` datasets, and train DeSpotX with three random seeds, with and without the diffusion prior. All other hyperparameters are held fixed across paired runs so that the only difference is the diffusion contribution.

We focus on established, biologically validated markers expressed at low levels in their native cell-clusters, where any removal by a decontamination model represents over-correction of real signal. Specifically, we select markers with intrinsic mean count < 3 in their native cell-clusters. For `MERFISH_Brain`, this yields 12 brain markers including neurotransmitter enzymes (Th, Chat, Nos1), neuropeptides (Crh, Vip, Gal, Pdyn, Tac2), dopamine receptors (Drd1, Drd2), an MGE transcription factor (Sox6), and calretinin (Calb2), with native means in the range 0.4 to 2.7. For `Xenium_Breast`, this yields 6 breast immune and proliferation markers, including MKI67 in proliferating tumor cells, ITGAX and C1QA in macrophages and dendritic cells, and CD27, GZMB, CTLA4 in T cells, with native means in the range 0.1 to 2.1.

For each (cell, marker) pair where the cell is native to that marker and the input count is positive, we compute retention $r = \hat{n}_{i,g}/s_{i,g}$. We then report the per-marker change in retention $\Delta r = r_{\text{with}} - r_{\text{without}}$ between runs with and without the diffusion prior, in percentage points. Positive values indicate that the diffusion prior helps preserve more native signal at low expression levels.

Figure 6 shows the per-marker Δr on both datasets. On `MERFISH_Brain`, every one of the 12 markers shows positive Δr, with values from $+0.33$ to $+1.55$ percentage points. On `Xenium_Breast`, 4 of 6 markers show positive Δr and 2 are neutral, with the largest gains on MKI67 ($+0.34$) and ITGAX ($+0.23$). These results indicate that the diffusion prior helps preserve low-expression marker-gene signal.

bioRxiv preprint doi: https://doi.org/10.64898/2026.05.12.724704; this version posted May 14, 2026. The copyright holder for this preprint (which was not certified by peer review) is the author/funder, who has granted bioRxiv a license to display the preprint in perpetuity. It is made available under a CC-BY 4.0 International license.

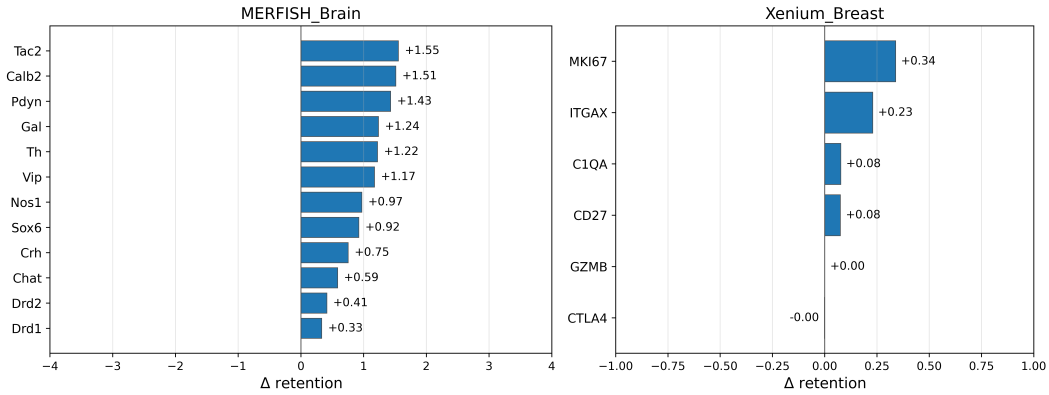

Figure 6: Effect of the diffusion prior on retention of low-but-real expression. Per-marker change in retention Δr between DeSpotX runs with and without the diffusion prior. Positive values indicate that the diffusion prior helps preserve more native signal.

G Robustness studies

We assess DeSpotX's robustness to perturbations of five inputs: the adaptive-anchor threshold κ, random perturbation of the anchor mask, noise in the cell-cluster labels, the number of spatial neighbors K, and the cell-cluster annotation method. For each factor, we run the spike-in benchmark on `Xenium_Breast` and `MERFISH_Brain` with the perturbed input while keeping the rest of the configuration at its default.

Robustness conditions. Each condition perturbs a single input from the default configuration.

- *Adaptive-anchor threshold*: κ is varied across $\{0.1, 0.2, 0.3, 0.4, 0.5\}$. Smaller κ produces a more permissive threshold and anchors more genes per cluster.
- *Anchor mask perturbation*: a uniform fraction $\{5\%, 10\%, 20\%\}$ of entries in the default $\kappa = 0.3$ anchor mask is randomly flipped (both $0 \to 1$ and $1 \to 0$).
- *Cell-cluster label perturbation*: the published cell-cluster annotation is perturbed by reassigning a random fraction $\{5\%, 10\%, 20\%, 30\%\}$ of cells to a different cluster, sampled uniformly. Both the cross-cluster mask in χ_i (Eq. 3) and the anchor mask (Eq. 20) are derived from the cluster annotation.
- *Number of spatial neighbors*: K is varied across $\{8, 12, 24\}$. K sets the number of spatial neighbors used by the GAT graph and the contamination estimator χ_i (Eq. 3).
- *Cell-cluster annotation method*: the published cell-cluster annotation is replaced by Leiden clustering at three target cluster counts $N \in \{15, 25, 30\}$. The Leiden resolution is binary-searched per dataset to hit each target.

Results. DeSpotX is robust to perturbations of the anchor mask. Across $\kappa \in \{0.1, 0.2, 0.3, 0.4, 0.5\}$, AUROC moves by at most 0.011 on either dataset, with PCE and GCE remaining close to their default values (Figure 7). When up to 20% of mask entries are flipped, no metric degrades on either dataset (Figure 8). DeSpotX does not require complete accuracy of the anchor mask, as identification can be supported by a sufficient subset of correct anchors within each cluster. This makes DeSpotX tolerant of moderate inaccuracies in the threshold κ and in the anchor mask.

Figure 9 shows that all three metrics remain close to the default values as the cell-cluster label perturbation rate increases from 0% to 30%. AUROC drops by less than 0.005 on both datasets, indicating that DeSpotX is not sensitive to label noise. Figure 10 shows that all three metrics remain close to the default values across $K \in \{8, 12, 24\}$ on both datasets, indicating that DeSpotX is robust to the choice of K in this range.

bioRxiv preprint doi: https://doi.org/10.64898/2026.05.12.724704; this version posted May 14, 2026. The copyright holder for this preprint (which was not certified by peer review) is the author/funder, who has granted bioRxiv a license to display the preprint in perpetuity. It is made available under a CC-BY 4.0 International license.

When the published cell-cluster annotation is replaced by Leiden clustering, AUROC drops by less than 0.03 on both datasets, while PCE and GCE remain close to the default values (Figure 11). The performance is consistent across the three Leiden cluster counts $N \in \{15, 25, 30\}$, indicating that DeSpotX is not sensitive to the specific cluster count chosen by Leiden.

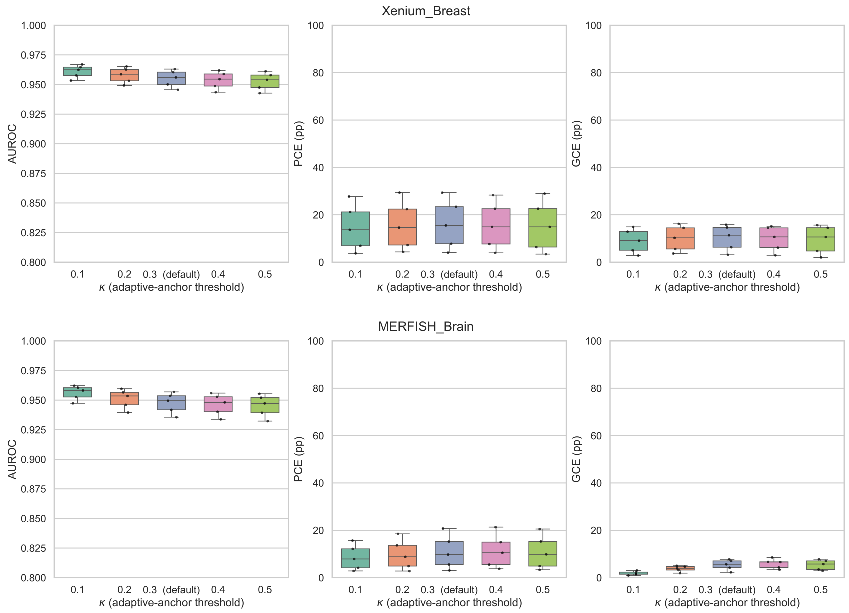

Figure 7: Robustness to the adaptive-anchor threshold κ. Each box summarizes the metric distribution across the five non-zero noise levels. PCE and GCE are reported in percentage points.

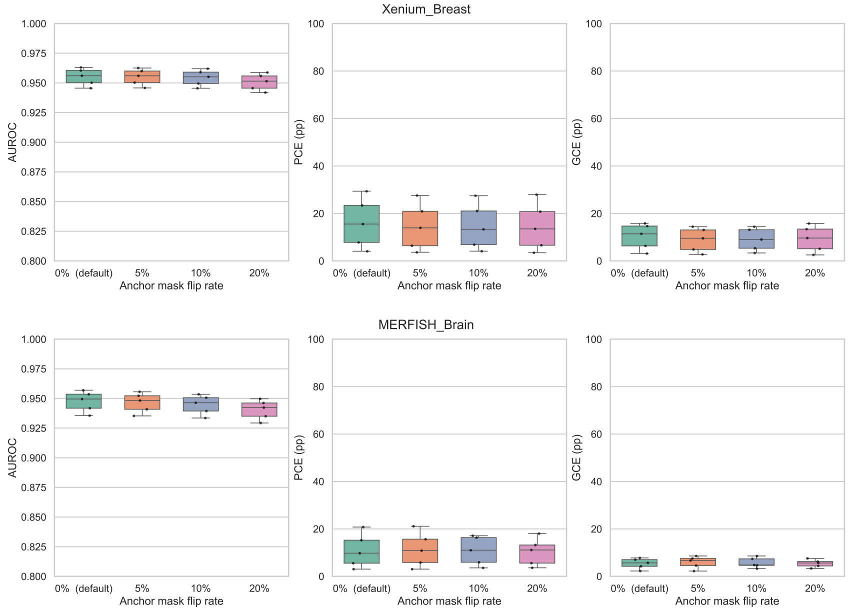

Figure 8: Robustness to random perturbation of the anchor mask. Each box summarizes the metric distribution across the five non-zero noise levels. PCE and GCE are reported in percentage points.

bioRxiv preprint doi: https://doi.org/10.64898/2026.05.12.724704; this version posted May 14, 2026. The copyright holder for this preprint (which was not certified by peer review) is the author/funder, who has granted bioRxiv a license to display the preprint in perpetuity. It is made available under a CC-BY 4.0 International license.

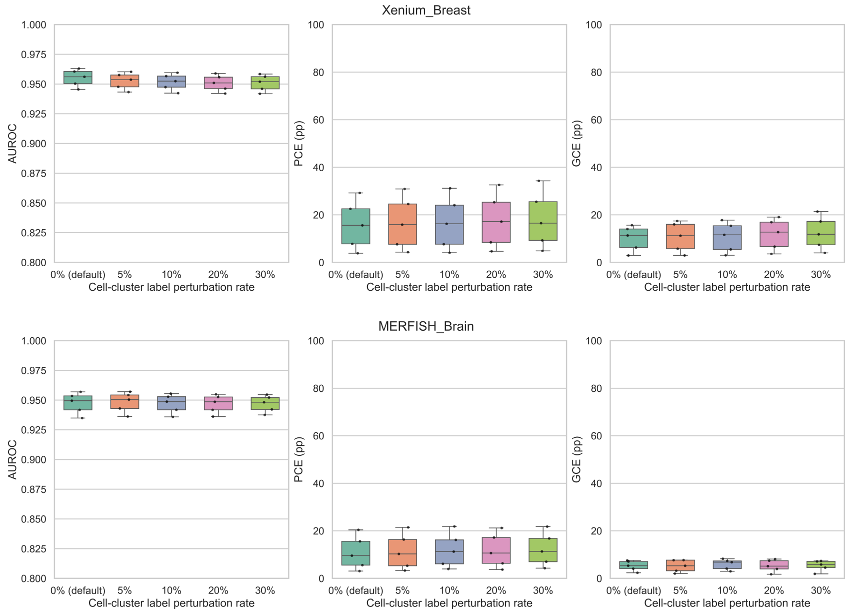

Figure 9: Robustness to cell-cluster label perturbation. Each box summarizes the metric distribution across the five non-zero noise levels. PCE and GCE are reported in percentage points.

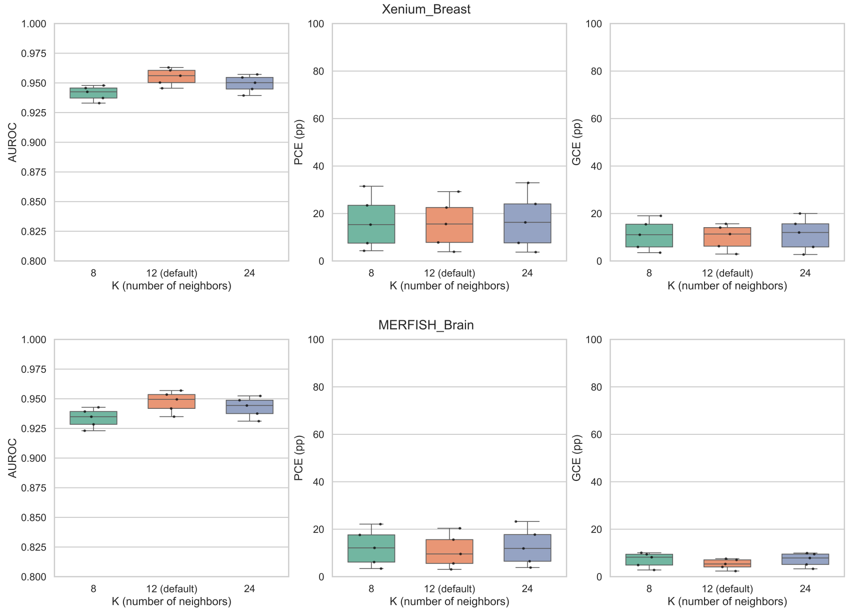

Figure 10: Robustness to the number of spatial neighbors K. Each box summarizes the metric distribution across the five non-zero noise levels. PCE and GCE are reported in percentage points.

bioRxiv preprint doi: https://doi.org/10.64898/2026.05.12.724704; this version posted May 14, 2026. The copyright holder for this preprint (which was not certified by peer review) is the author/funder, who has granted bioRxiv a license to display the preprint in perpetuity. It is made available under a CC-BY 4.0 International license.

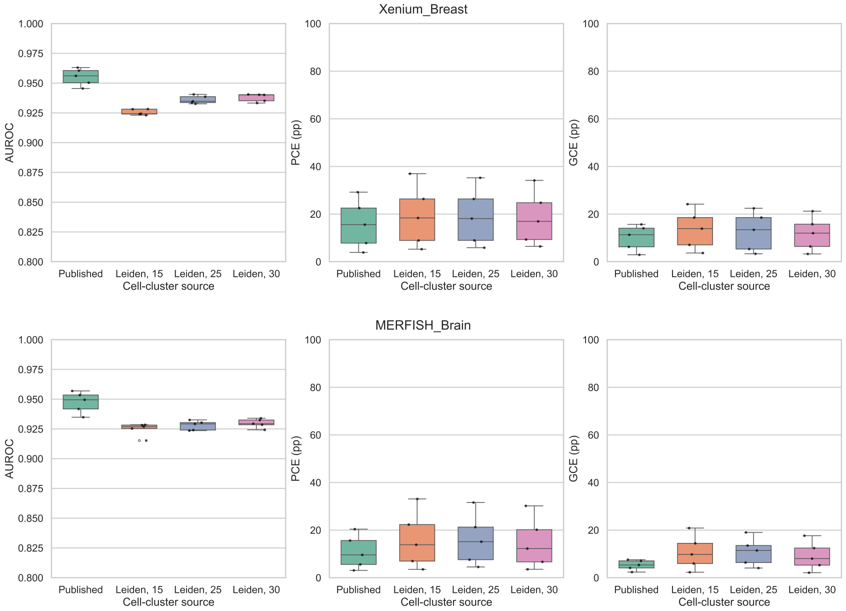

Figure 11: Robustness to the cell-cluster annotation method. Each box summarizes the metric distribution across the five non-zero noise levels. PCE and GCE are reported in percentage points.

H Effect of contamination level on performance

We examine how DeSpotX's performance varies with the noise level ε on each of the five spike-in benchmark datasets.

AUROC of DeSpotX across noise levels. Figure 12 shows DeSpotX's AUROC at each noise level on each dataset. Across all five datasets, AUROC varies by at most 0.025 between $\bar{\varepsilon} = 5\%$ and $\bar{\varepsilon} = 40\%$. On `Xenium_Breast` and `MERFISH_Brain`, AUROC increases slightly with $\bar{\varepsilon}$, since stronger contamination produces clearer signals that are easier to identify against the native expression background. DeSpotX therefore performs consistently across contamination levels.

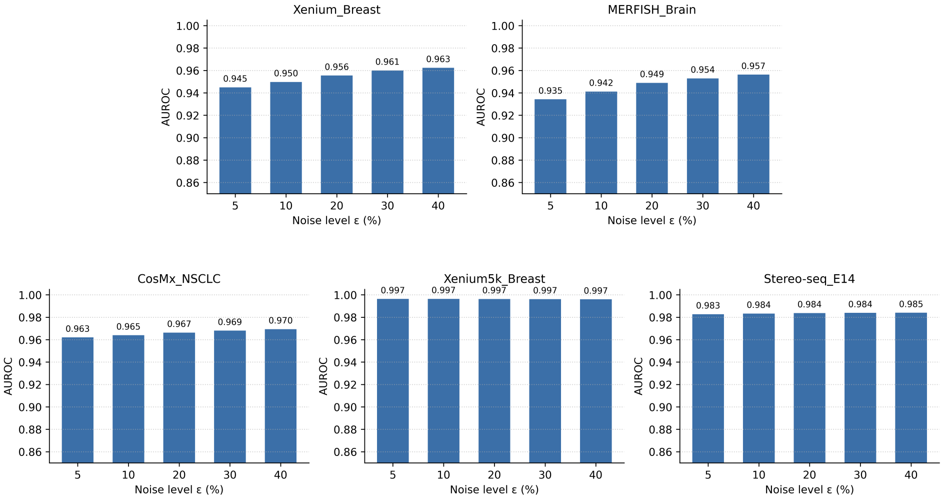

Figure 12: DeSpotX's AUROC on each of the five spike-in benchmark datasets at five contamination levels $\varepsilon \in \{5, 10, 20, 30, 40\}\%$.

bioRxiv preprint doi: https://doi.org/10.64898/2026.05.12.724704; this version posted May 14, 2026. The copyright holder for this preprint (which was not certified by peer review) is the author/funder, who has granted bioRxiv a license to display the preprint in perpetuity. It is made available under a CC-BY 4.0 International license.

Per-cell calibration advantage of DeSpotX across noise levels. We compare DeSpotX against DecontX and ResolVI as representative baselines for scRNA-seq and ST methods, respectively. Figure 13 reports the per-cell calibration error gap $\Delta\text{PCE} = \text{PCE}_{\text{baseline}} - \text{PCE}_{\text{DeSpotX}}$ between DeSpotX and these two baselines, in percentage points. Positive values indicate that DeSpotX achieves lower calibration error than the baseline. On every dataset and at every $\bar{\varepsilon} \geq 10\%$, both gaps are positive, and the gaps grow with $\bar{\varepsilon}$. At $\bar{\varepsilon} = 5\%$, the gaps are within 3 pp; at $\bar{\varepsilon} = 40\%$, the gaps reach 11–13 pp on `CosMx_NSCLC` and `MERFISH_Brain` and 22–27 pp on `Xenium5k_Breast`. The increasing gap shows that DeSpotX's per-cell contamination estimates remain accurate as the contamination signal grows, while baseline calibration error scales with the contamination level.

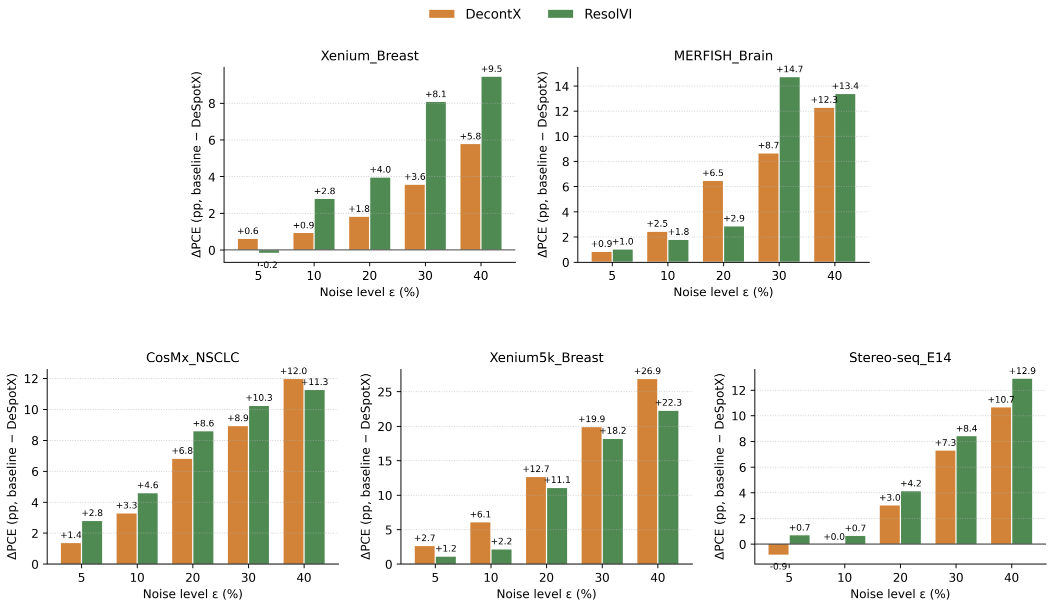

Figure 13: Per-cell calibration error gap $\Delta\text{PCE} = \text{PCE}_{\text{baseline}} - \text{PCE}_{\text{DeSpotX}}$ between DeSpotX and the representative baselines (DecontX, ResolVI), in percentage points, at five contamination levels $\bar{\varepsilon} \in \{5, 10, 20, 30, 40\}\%$.

I Validation of the within-cluster bound

We test Lemma 3 (Appendix B.2) on a spike-in simulation in which each cell receives contamination from a mixture of its cross-cluster and same-cluster spatial neighbors, weighted α and $1 - \alpha$ respectively, with per-cell rate sampled from a Beta distribution centered at $\bar{\varepsilon} = 0.2$. We vary $\alpha \in \{0.3, 0.5, 0.7, 0.9, 1.0\}$ on `Xenium_Breast` and `MERFISH_Brain`.

AUROC on cross-cluster contamination labels stays above 0.93 across all α on both datasets (Figure 14a), confirming $\hat{\varepsilon}_i$ tracks the cross-cluster rate. The per-cell native-profile error grows linearly with the scaling factor β_i, with slopes 0.015 on `Xenium_Breast` and 0.010 on `MERFISH_Brain` (Figure 14b), well below the empirical $\langle \sigma^{\text{within}} \rangle$ of 0.057 and 0.063 measured across the five datasets (Figure 14c). The intercept (≈ 0.022) reflects α-independent error sources unrelated to within-cluster contamination, including DeSpotX's removal of natural platform contamination from the unspiked reference.

bioRxiv preprint doi: https://doi.org/10.64898/2026.05.12.724704; this version posted May 14, 2026. The copyright holder for this preprint (which was not certified by peer review) is the author/funder, who has granted bioRxiv a license to display the preprint in perpetuity. It is made available under a CC-BY 4.0 International license.

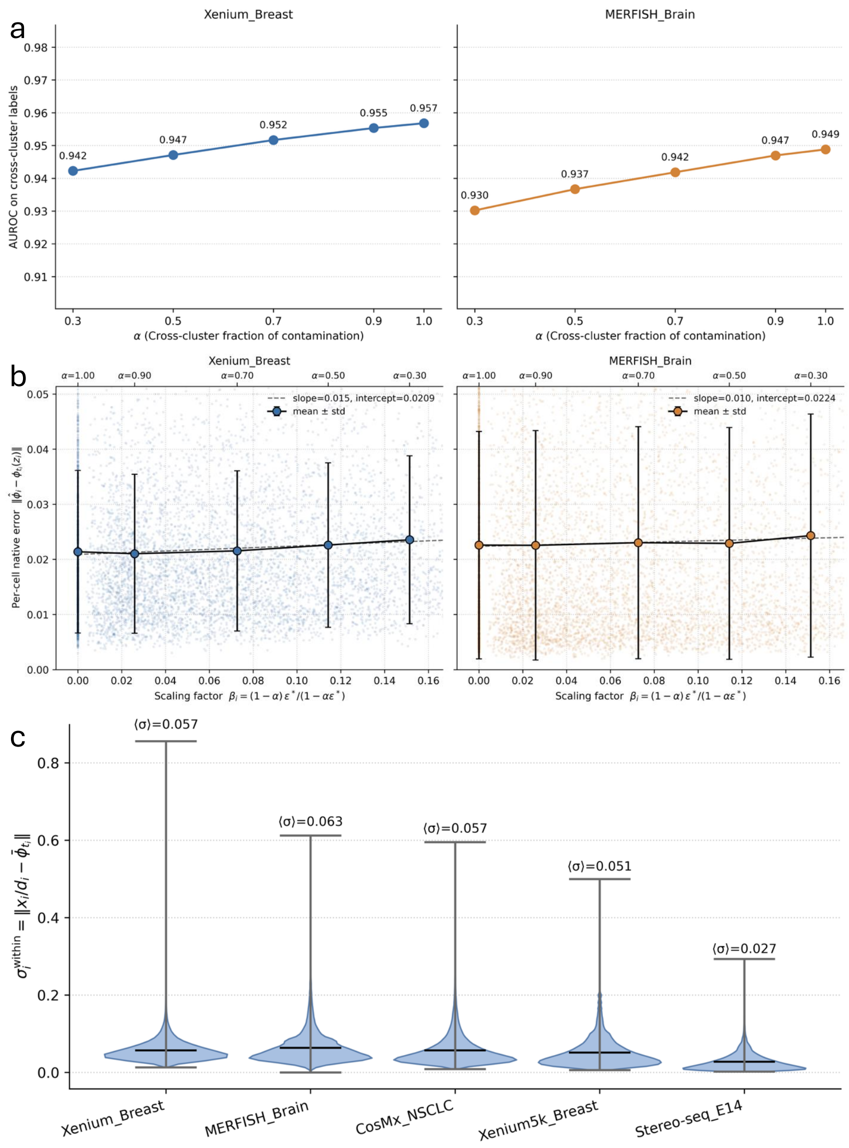

Figure 14: DeSpotX is robust to within-cluster contamination across spike-in mixtures. (a) AUROC for distinguishing cross-cluster contamination from native counts, across $\alpha \in \{0.3, 0.5, 0.7, 0.9, 1.0\}$. (b) Per-cell native-profile error $\|\hat{\phi}_i - \phi_{t_i}(z_i)\|$ versus the scaling factor $\beta_i = (1-\alpha)\varepsilon^*/(1-\alpha\varepsilon^*)$. Markers show mean $\pm$ std per α; dashed line is a linear fit. (c) Distribution of σ_i^{within} across cells in all five datasets at zero injected contamination, with mean values annotated.

J Runtime comparison

We compare end-to-end runtime across decontamination methods on `Xenium5k_Breast` (577,258 cells, 5,101 genes) and `Stereo-seq_E14` (92,928 cells, 18,582 genes), the two largest datasets used in this work (Figure 15). SoupX and DecontX run in 3 to 10 minutes. Among the deep learning-based methods, DeSpotX is the fastest at 16 to 21 minutes. ResolVI and SpaceBender take 6 to 53 hours. All methods were run on a single NVIDIA Tesla T4 GPU (16 GB).

bioRxiv preprint doi: https://doi.org/10.64898/2026.05.12.724704; this version posted May 14, 2026. The copyright holder for this preprint (which was not certified by peer review) is the author/funder, who has granted bioRxiv a license to display the preprint in perpetuity. It is made available under a CC-BY 4.0 International license.

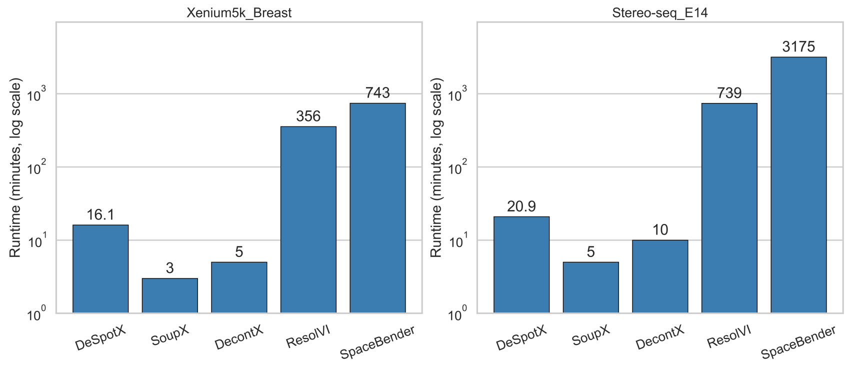

Figure 15: Runtime comparison across decontamination methods on `Xenium5k_Breast` and `Stereo-seq_E14`.

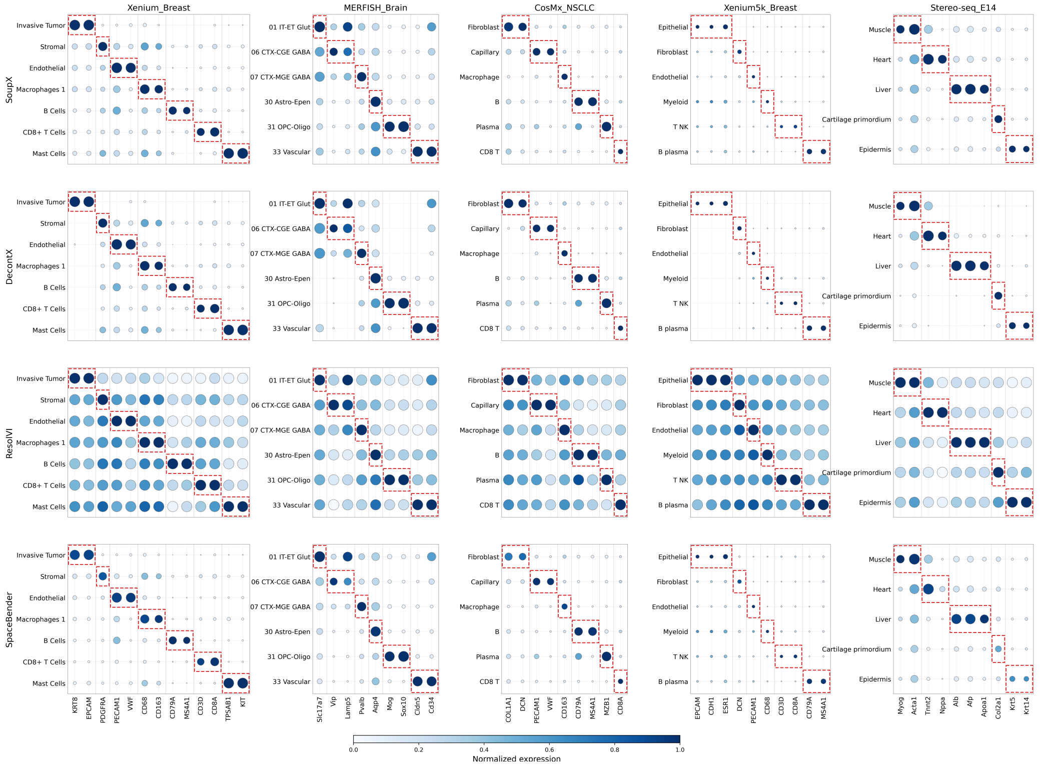

Figure 16: Marker-gene dotplots for the four baseline methods across the five datasets. Red dashed boxes mark canonical marker-cluster pairs.

bioRxiv preprint doi: https://doi.org/10.64898/2026.05.12.724704; this version posted May 14, 2026. The copyright holder for this preprint (which was not certified by peer review) is the author/funder, who has granted bioRxiv a license to display the preprint in perpetuity. It is made available under a CC-BY 4.0 International license.

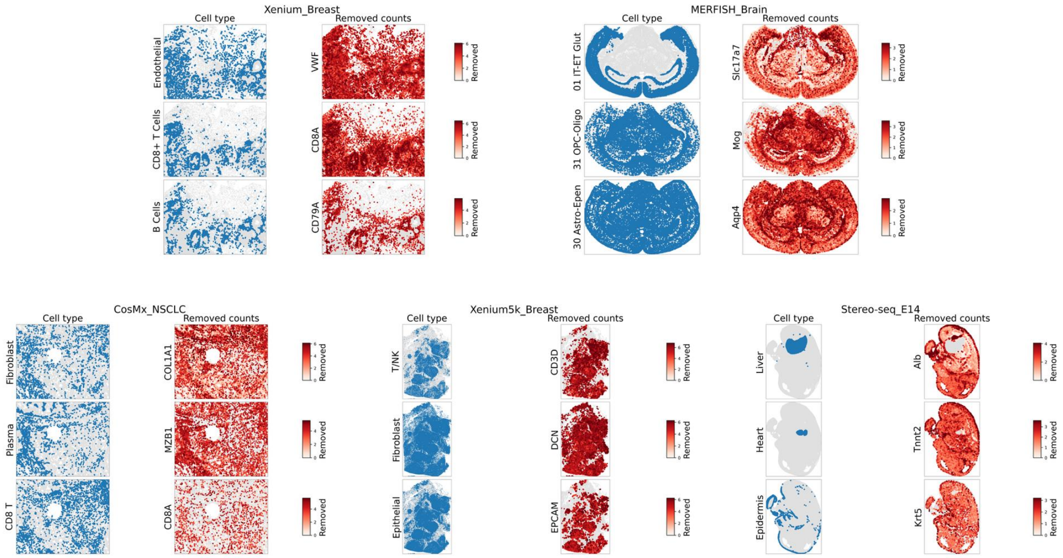

Figure 17: Spatial maps of DeSpotX-decontaminated counts for canonical cell-cluster markers across the five datasets. For each marker, cells of the canonical expressing cluster are highlighted on the left, and the spatial distribution of removed counts is shown on the right.

K Marker-gene specificity of baseline methods

We extend the marker-gene comparison in Figure 2b to the four baseline methods (Figure 16). SoupX produces dotplots that closely resemble the raw counts, with off-target expression largely unchanged. DecontX and SpaceBender reduce off-target expression to varying degrees across datasets, but residual signal in non-canonical clusters remains visible, particularly on the larger panels (Xenium5k_Breast, CosMx_NSCLC). ResolVI shows little difference between canonical and non-canonical clusters. All four baselines yield lower marker-gene specificity than DeSpotX.

L Spatial localization of removed contamination

To examine where DeSpotX removes contamination, we map the per-cell amount removed for canonical markers of representative cell clusters across the five datasets (Figure 17). The removed counts are spatially concentrated near the canonical expressing cluster, consistent with the expected pattern of ambient contamination, in which a marker's signal diffuses from cells that natively express it into nearby cells, and DeSpotX removes the diffused signal from the receiving cells. For example, VWF in `Xenium_Breast` is endothelial-specific and Mog in `MERFISH_Brain` is oligodendrocyte-specific; in both cases, DeSpotX's removal extends beyond the canonical cluster into surrounding tissue, with the highest removal in regions immediately adjacent to the canonical cells. This pattern holds across the five datasets and supports the spatial-locality assumption underlying DeSpotX's contamination model.

M Spatial coherence of baseline methods

We extend the Moran's I comparison in Figure 3 to the four baseline methods (Figure 18). SoupX and DecontX produce scatters that closely follow the identity line, indicating that their decontamination preserves the raw spatial structure of expression but does not enhance it. SpaceBender's scatters fall on or below the diagonal across all five datasets, suggesting that its decontamination reduces spatial coherence relative to the raw counts. ResolVI shows substantially elevated Moran's I values across all five datasets, often saturating at 0.4–0.8 even for genes with near-zero raw values. This pattern reflects ResolVI's spatial-smoothing decoder, which incorporates neighbor information into each cell's expression estimate and inflates Moran's I regardless of whether the underlying signal is

bioRxiv preprint doi: https://doi.org/10.64898/2026.05.12.724704; this version posted May 14, 2026. The copyright holder for this preprint (which was not certified by peer review) is the author/funder, who has granted bioRxiv a license to display the preprint in perpetuity. It is made available under a CC-BY 4.0 International license.

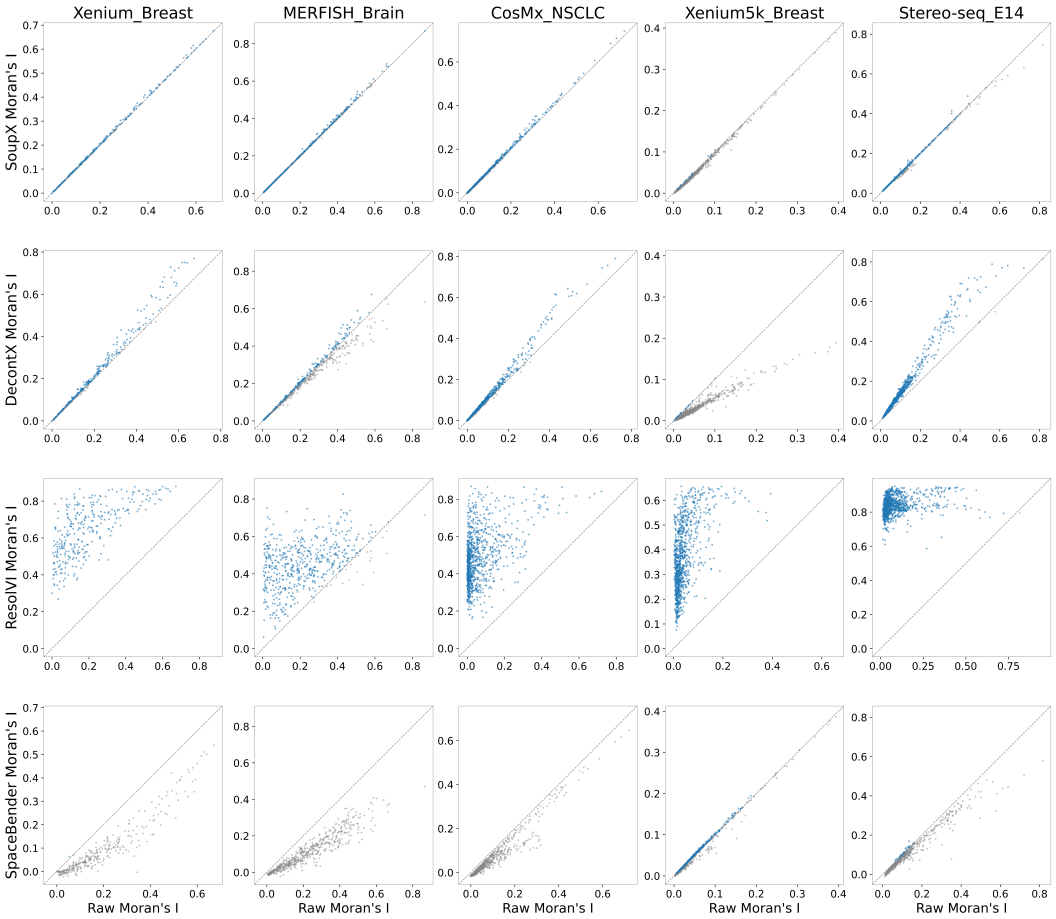

Figure 18: Per-gene Moran's I for raw and decontaminated counts from each baseline method (SoupX, DecontX, ResolVI, SpaceBender) across the five datasets. Each panel plots the raw Moran's I on the x-axis against the decontaminated Moran's I on the y-axis; the dashed line denotes equality.

biological or ambient. Such smoothing produces visually coherent maps but does not separate native signal from ambient contamination. By contrast, DeSpotX (Figure 3) raises Moran's I above the diagonal proportionally to the raw value, indicating that improvements arise from removing local contamination rather than from spatial averaging.

N Iterative decontamination on Xenium_Breast

We apply the iterative procedure described in Section 4.3.3 to the `Xenium_Breast` dataset. The CellTypist classifier [22] for this dataset was trained on a published single-cell breast cancer atlas and covers nine broad cell-cluster categories spanning normal and malignant epithelial populations.

The cluster-validity metrics improve significantly from iteration 0 to iteration 1 and stabilize thereafter (Figure 19a), with Silhouette rising from 0.07 to 0.19 and Calinski-Harabasz increasing from $1{,}590$ to $2{,}089$. The ARI between consecutive iterations approaches 1.0 by iteration 2, indicating that the procedure converges to a stable annotation. Additionally, the UMAP embeddings show clearer separation of breast cell types across iterations (Figure 19b).

CellChat [25] inference shows a reduction in the number of significant interactions between iteration 0 and iteration 1, after which the network stabilizes (Figure 19c). The reduction is largely driven by removal of interactions involving the secreted ligand PTN (pleiotrophin). At iteration 0, PTN is inferred to be sent from cancer (DCIS_2) and endothelial cells; by iteration 5, these interactions are

bioRxiv preprint doi: https://doi.org/10.64898/2026.05.12.724704; this version posted May 14, 2026. The copyright holder for this preprint (which was not certified by peer review) is the author/funder, who has granted bioRxiv a license to display the preprint in perpetuity. It is made available under a CC-BY 4.0 International license.

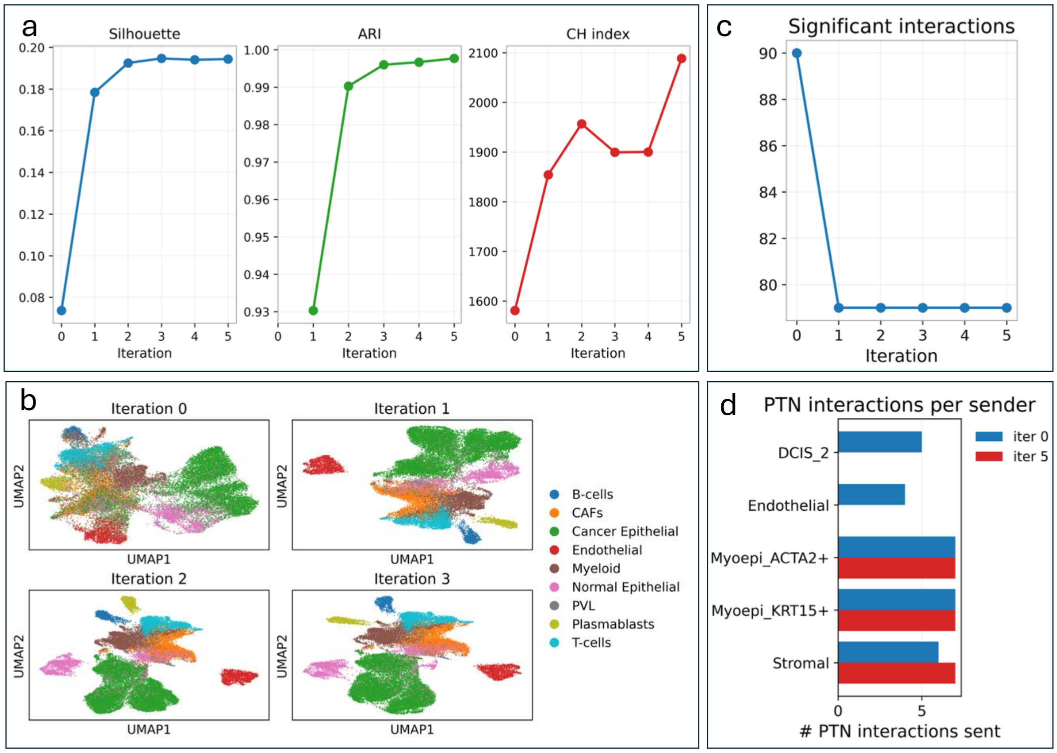

Figure 19: Iterative DeSpotX decontamination and cell-cluster annotation on `Xenium_Breast`. (a) Cluster-validity metrics across iterations. (b) UMAP embeddings of the decontaminated counts at iterations 0-3. (c) Total number of significant cell-cell interactions inferred by CellChat across iterations. (d) Number of significant PTN-pathway interactions per sender cell-cluster at iteration 0 and iteration 5.

removed, while PTN signaling from myoepithelial cells, which natively express PTN in mammary tissue, is preserved (Figure 19d).
